# A Terrylene–Anthraquinone Dyad as a Chromophore for Photothermal Therapy in the NIR-II Window

**DOI:** 10.1021/jacs.3c11314

**Published:** 2023-11-27

**Authors:** Ze-Hua Wu, Min Peng, Chendong Ji, Panagiotis Kardasis, Ioannis Tzourtzouklis, Martin Baumgarten, Hao Wu, Thomas Basché, George Floudas, Meizhen Yin, Klaus Müllen

**Affiliations:** † Max Planck Institute for Polymer Research, Ackermannweg 10, Mainz 55128, Germany; ‡ State Key Laboratory of Chemical Resource Engineering, Beijing Laboratory of Biomedical Materials, Beijing University of Chemical Technology, Beijing 100029, China; § Department of Chemistry, Johannes Gutenberg-University, Mainz 55099, Germany; # Department of Physics, University of Ioannina, Ioannina 45110, Greece; ∇ University Research Center of Ioannina (URCI) - Institute of Materials Science and Computing, Ioannina 45110, Greece

## Abstract

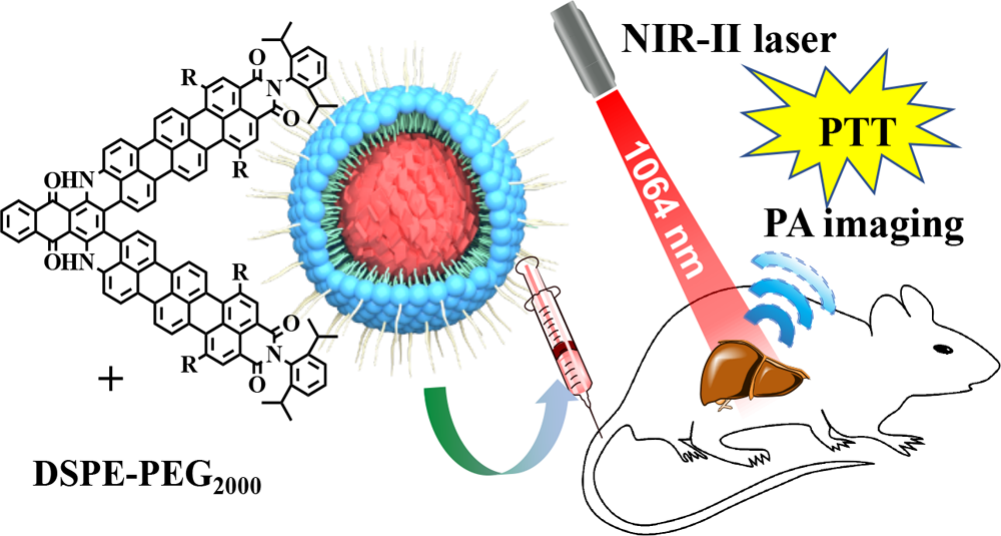

A terrylenedicarboximide–anthraquinone dyad, **FTQ**, with absorption in the second near-infrared region (NIR-II) is obtained as a high-performance chromophore for photothermal therapy (PTT). The synthetic route proceeds by C–N coupling of amino-substituted terrylenedicarboximide (TMI) and 1,4-dichloroanthraquinone followed by alkaline-promoted dehydrocyclization. **FTQ** with extended π-conjugation exhibits an optical absorption band peaking at 1140 nm and extending into the 1500 nm range. Moreover, as determined by dielectric spectroscopy in dilute solutions, **FTQ** achieves an ultrastrong dipole moment of 14.4 ± 0.4 Debye due to intense intramolecular charge transfer. After encapsulation in a biodegradable polyethylene glycol (DSPE-mPEG2000), **FTQ** nanoparticles (NPs) deliver a high photothermal conversion efficiency of 49% under 1064 nm laser irradiation combined with excellent biocompatibility, photostability, and photoacoustic imaging capability. *In vitro* and *in vivo* studies reveal the great potential of **FTQ** NPs in photoacoustic-imaging-guided photothermal therapy for orthotopic liver cancer treatment in the NIR-II window.

## Introduction

Photothermal therapy (PTT) is one of the most advanced technologies in nanomedicine that employs laser irradiation to generate localized heat for ablating cancer cells.^1,2^ PTT holds promise in treating critical diseases such as hepatocellular carcinoma (HCC), which is the most common primary liver cancer and the third leading lethal cancer.^3^ At present, the conventional treatments are chemotherapy, radiotherapy, and surgical resection, which, however, cannot avoid normal organ damage and acute and/or late systemic toxicity.^4^ In contrast, PTT offers the advantages of high efficiency and minimal invasion of normal tissue. Moreover, PTT is promising for both treatment and diagnosis of HCC.^5,6^ So far, PTT studies have concentrated on the first near-infrared region (NIR-I, 700–1000 nm), although the second near-infrared region (NIR-II, 1000–1700 nm) is emerging as a more preferable optical window owing to minimized photodamage to healthy tissue, deeper penetration, and less energy dissipation.^7,8^

Compared with inorganic absorbers,^9^ organic chromophores have advantages in view of versatile structures, long-term safety, and biocompatibility.^10−12^ Currently, the availability of organic NIR-II absorbers is still limited and mainly based on established scaffolds such as benzo[1,2-c:4,5-c′]bis[1,2,5]thiadiazole (BBTD),^13^ dipyrrometheneboron difluoride (BODIPY),^14^ and phthalocyanines.^15^ In addition, stability is an important issue that has compromised the bioapplication of most organic NIR-II chromophores.^10,16^ Thus, it is essential to explore new organic NIR-II absorbers as efficient PTT agents.

**Figure 1:**
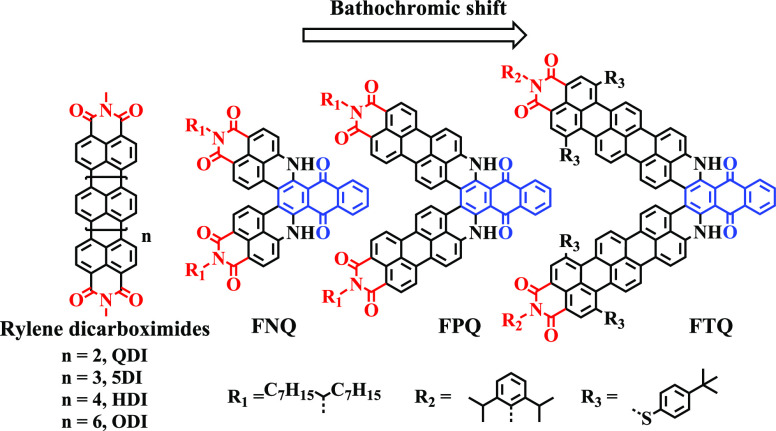
Molecular structure of rylene dicarboximides, **FNQ**, **FPQ**, and **FTQ**.

Rylene dicarboximides (Figure 1) are considered as a promising candidate due to their exceptional stability and high photothermal conversion efficiency (PCE).^17^ In our recent study,^18^ we have introduced a series of novel NIR chromophores based on anthraquinone fused with naphthalene-1,8-dicarboximide (**FNQ**) and perylene-3,4-dicarboximide (**FPQ**), which demonstrate maximum absorption peaks (λ_max_) above 720 nm. However, to achieve the PTT effect in the NIR-II window, further improvement of the absorption domain and processability is urgently required. Extending the conjugation to terrylene-3,4-dicarboximide (TMI) appears to be the logical next step to narrow the optical gap and induce a further bathochromic shift. Herein, we introduce a dyad of bay-position-substituted TMI and anthraquinone as a new NIR-II absorber. The synthetic route utilizes an efficient C–N coupling of amino-substituted TMI with 1,4-dichloroanthraquinone followed by alkaline-promoted dehydrocyclization. **FTQ** exhibits a maximum absorption band at 1140 nm, with the tail extending to 1500 nm, deep into the NIR-II region. Moreover, **FTQ** presents a dipole moment of 14.4 ± 0.4 Debye, demonstrating a new type of charge-free organic molecule with a ultrastrong dipole moment.^19^**FTQ** nanoparticles (NPs) prepared by assembling with 1,2-distearoyl-*sn*-glycero-3-phosphoethanolamine-*N*-[methoxy-(polyethylene glycol)-2000] (DSPE-PEG2000) demonstrate good photostability and biocompatibility combined with a high photothermal conversion efficiency of 49% under 1064 nm laser irradiation. Remarkably, *in vivo* studies reveal an excellent tumor ablation performance of **FTQ** NPs, allowing photothermal therapy for orthotopic liver cancer treatment in the NIR-II window.

## Results and Discussion

**Scheme 1:**
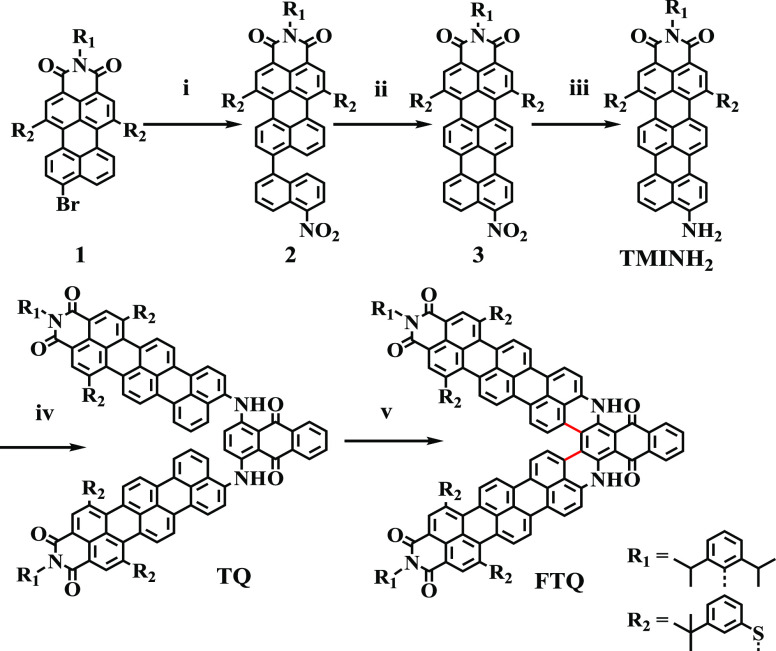
Synthetic Route toward **FTQ** Reagents and conditions: (i) Pd(PPh_3_)_4_, K_2_CO_3_, 4,4,5,5-tetramethyl-2-(5-nitronaphthalen-1-yl)-1,3,2-dioxaborolane, H_2_O/ethanol/toluene, 80 °C, 16 h, 80%; (ii) tBuONa, DBN, diglyme, 70 °C, 2 h, 70%; (iii) Pd/C, H_2_, ethanol/CHCl_3_, r.t., 15 h, 78%; (iv) BrettPhos Pd G1, BrettPhos, Cs_2_CO_3_, toluene, 110 °C, 12 h, 70%; (v) tBuONa, DBN, diglyme, 130 °C, 16 h, 60%.

As depicted in Scheme 1, the synthetic route consisted of two parts: the construction of the **TMINH**_**2**_ building block and **FTQ**. **TMINH**_**2**_ was constructed through three steps: C–C coupling, dehydrocyclization, and reduction. Suzuki coupling of **1** and 4,4,5,5-tetramethyl-2-(5-nitronaphthalen-1-yl)-1,3,2-dioxaborolane afforded **2** in 80% yield. The terrylene skeleton was constructed through an alkali-induced dehydrocyclization reaction with sodium *tert*-butoxide (tBuONa) and 1,5-diazabicyclo[4.3.0]non-5-ene (DBN) as the base in 70% yield. The nitro group of **3** was then reduced to the amino substituent through hydrogenation over Pd/C, producing **TMINH**_**2**_ in 78% yield. Herein, 4-*tert*-butylbenzenethiol was selected as the substituent at the bay region of the TMI to prevent aggregation. In comparison with phenol substituents, benzenethiol is able to react at room temperature and further promote a bathochromic shift of the absorption.^20^ Afterward, a C–N coupling connected **TMINH**_**2**_ and 1,4-dichloroanthraquinone to produce **TQ** by using 2-(dicyclohexylphosphino)3,6-dimethoxy-2,4,6′-triisopropyl-1,1′-biphenyl (BrettPhos) Pd G1 as catalyst and cesium carbonate as base, furnishing **TQ** in 70% yield. To fuse anthraquinone at the peri-positions of TMI in **TQ**, a cyclodehydrogenation reaction was conducted as the key step. The commonly used oxidative cyclodehydrogenation reaction with FeCl_3_ or DDQ failed,^21^ which can be explained by the electron-deficient nature of the TMI and anthraquinone entities. Therefore, we employed alkaline-promoted dehydrocyclization with tBuONa and DBN as an alternative ring fusion reaction to produce **FTQ** in 60% yield.

**Figure 2:**
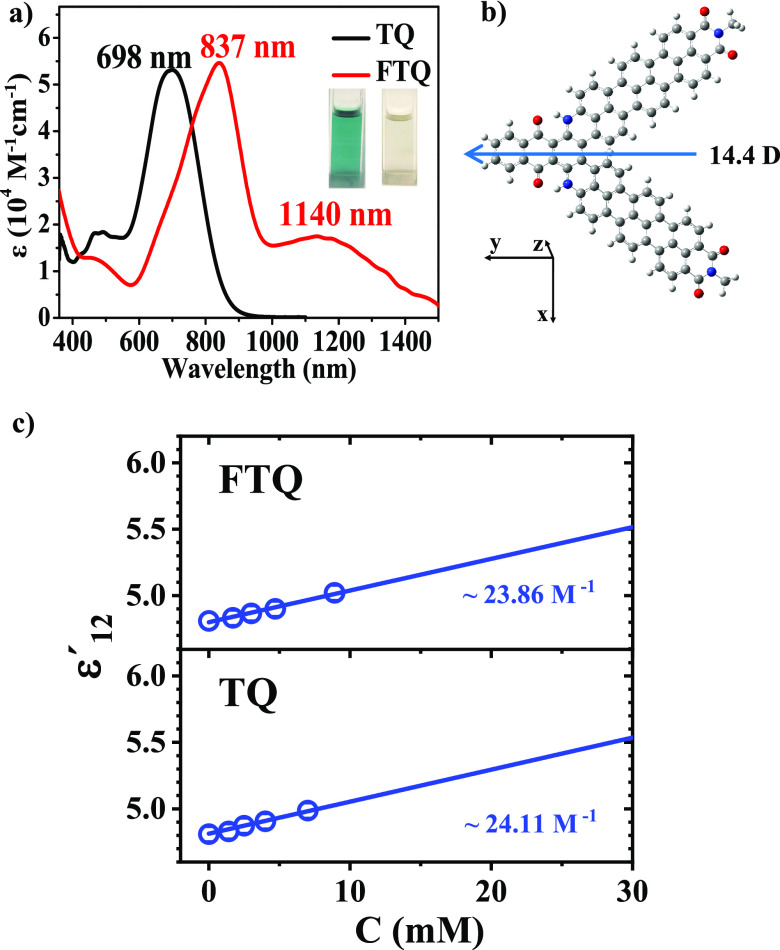
(a) Absorption spectra of **TQ** and **FTQ**. Inset: photographs of **TQ** (left) and **FTQ** (right) solutions (1 × 10^–5^ M in dichloromethane). (b) Optimized structure and dipole moment determined by dielectric spectroscopy. (c) Dielectric permittivity of **FTQ** (top) and **TQ** (bottom) as a function of concentration in chloroform solutions. The highest concentrations refer to the solubility limits of each compound. The electric dipole moments were calculated from the slopes of the linear fits. The uncertainty is smaller than the symbol size.

The UV–vis-NIR absorption spectrum of **FTQ** is depicted in Figure 2a. Compared with precursor **TQ**, **FTQ** revealed a pronounced bathochromic shift of 442 nm, reflecting the significantly extended π-conjugation after dehydrocyclization. Importantly, in comparison with the NMI (λ_max_ = 872 nm) and PMI fused analogues (λ_max_ = 1028 nm),^18^ extending the conjugation to **FTQ** induced further red-shifted absorption, displaying λ_max_ at 1140 nm and an intense absorption peak (λ_1_) at 837 nm with molar absorption coefficients of 17,628 and 54,833 M^–1^cm^–1^, respectively.

Density functional theory (DFT) calculation of **FTQ** at the B3LYP/6-31 G* level revealed a strong dipole moment of 13.1 Debye along the *y* axis (Figure 2b). The dipole moment is pointing from the TMI moieties toward anthraquinone, indicating an intense intramolecular charge transfer (ICT)^22^ between the two components. The electric dipole moments of **FTQ** and **TQ** were experimentally determined by using dielectric spectroscopy. The complex dielectric permittivity ε* = ε’ – iε″ (where ε’ is the real and ε″ is the imaginary part) was measured as a function of the solute concentration in chloroform. Employing the modified Onsager equation according to Böttcher and assuming ideal solutions of the two components, the dipole moment of the solute can be obtained from the derivative of the real part of the measured dielectric permittivity, ε_12_, with respect to the concentration at the limit of infinite dilution.^19,23−25^ Details are described in the Supporting Information.

The measured dielectric permittivity as a function of the concentration is shown in Figure 2c. The dipole moment of **FTQ** was calculated to be 14.4 ± 0.4 Debye. Charge-free organic molecules seldom achieve dipole moments above 10 Debye. **FTQ** thus displays a new type of molecular design toward ultrastrong dipole moments desirable in ferroelectrics and organic photovoltaics.^19^ The precursor **TQ** also exhibited an ultrastrong dipole moment of 14.0 ± 0.6 Debye, which confirmed the strong ICT.

**Figure 3:**
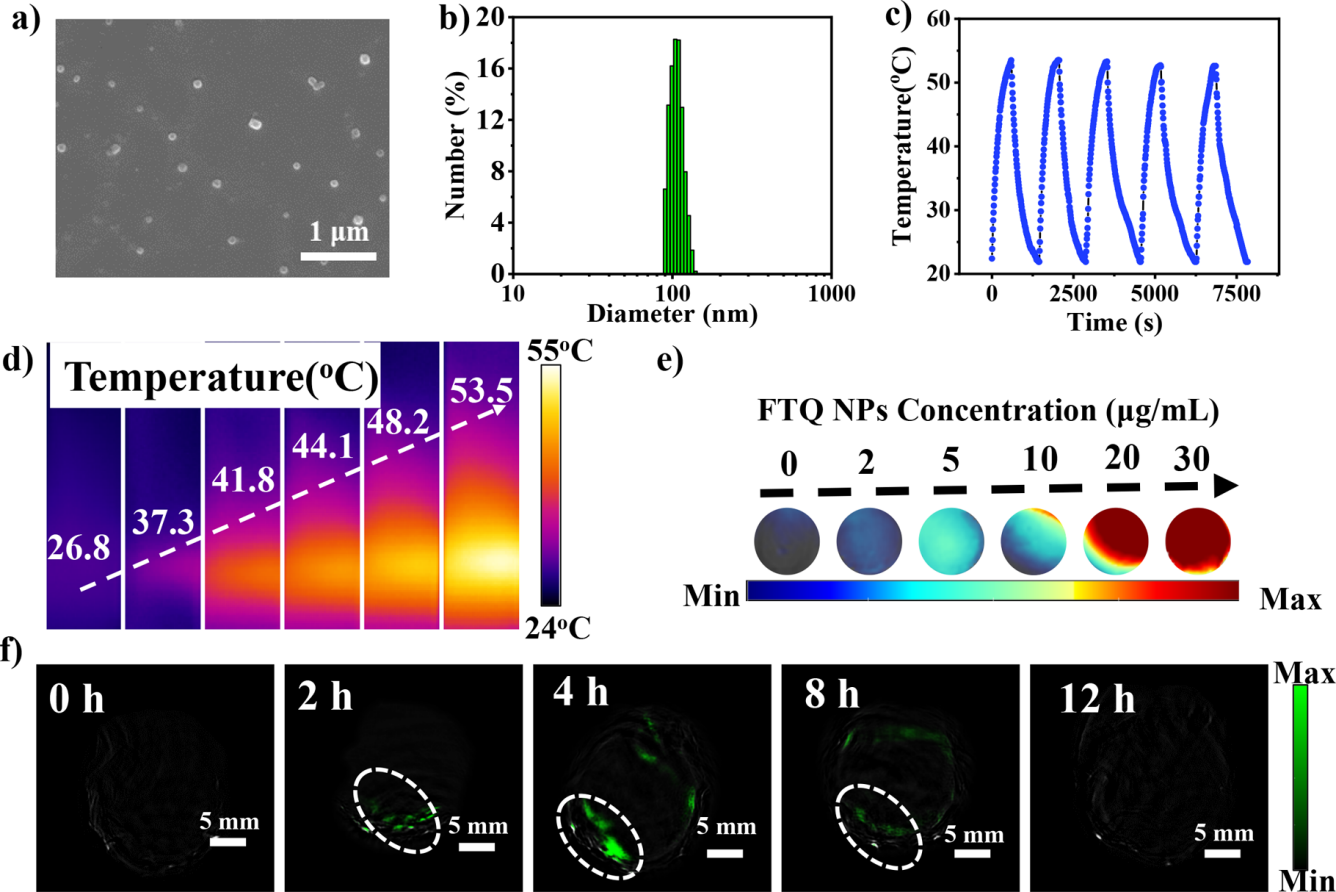
(a) TEM image of **FTQ** NPs. (b) DLS profile of **FTQ** NPs. (c) Photothermal stability of **FTQ** NPs upon laser irradiation (1064 nm, 1.0 W cm^–2^) after five on/off cycles. (d) Thermal image of **FTQ** NP solutions after laser irradiation for 10 min (concentrations from left to right: 0, 2, 5, 10, 20, and 30 μg/mL). (e) *In vitro* PA images of **FTQ** NPs at different concentrations. (f) *In vivo* PA images of **FTQ** NPs in orthotopic liver tumor tissue at different times.

**FTQ** nanoparticles (NPs) with high water solubility (>12 mg/mL) were prepared with DSPE-PEG2000 as the encapsulation matrix.^26^ The morphology and size of **FTQ** NPs were investigated by transmission electron microscopy (TEM) and dynamic light scattering (DLS) at 25 °C. As shown in Figure 3a,b, **FTQ** NPs presented uniform self-assembled spherical NPs with an average diameter of 110.8 ± 0.8 nm, which were suitable for achieving an enhanced permeability and retention (EPR) effect. After storage at 4 °C for 2 weeks, **FTQ** NPs manifested high stability in phosphate-buffered saline (PBS) without obvious change in morphology and absorption (Figure S8), which was essential for long-term circulation *in vivo*.

**FTQ** displayed negligible emission and the generation of reactive oxygen species (Figure S9). This points toward nonradiative vibrational relaxation as a dominant factor in excited-state deactivation and as the reason for efficient photothermal conversion.^17^ The PCE of **FTQ** NPs was investigated under NIR-II laser (1064 nm) irradiation that possesses deeper tissue penetration (>2 cm) and higher maximum permissible exposure (1.0 W cm^2^) than the NIR-I light source. The PCE (η)^27,28^ of **FTQ** NPs was calculated to be 49%, which is higher than those of most NIR-II PPT agents such as boron difluoride formazanate^1^ and benzo[1,2-c:4,5-c’]bis([1,2,5]thiadiazole).^13^**FTQ** NPs exhibited excellent thermal stability and photostability following five heating and cooling cycles under laser irradiation (Figure 3c). In addition, NIR light irradiation of **FTQ** NPs resulted in a photoacoustic (PA) signal in the phantom mold (Figure 3e). As demonstrated in Figure S10e, a linear relationship between the concentration and the average PA signal was achieved (coefficient of determination, *R*^2^ = 0.99), supporting the role of **FTQ** NPs as PA imaging agents.

**Figure 4:**
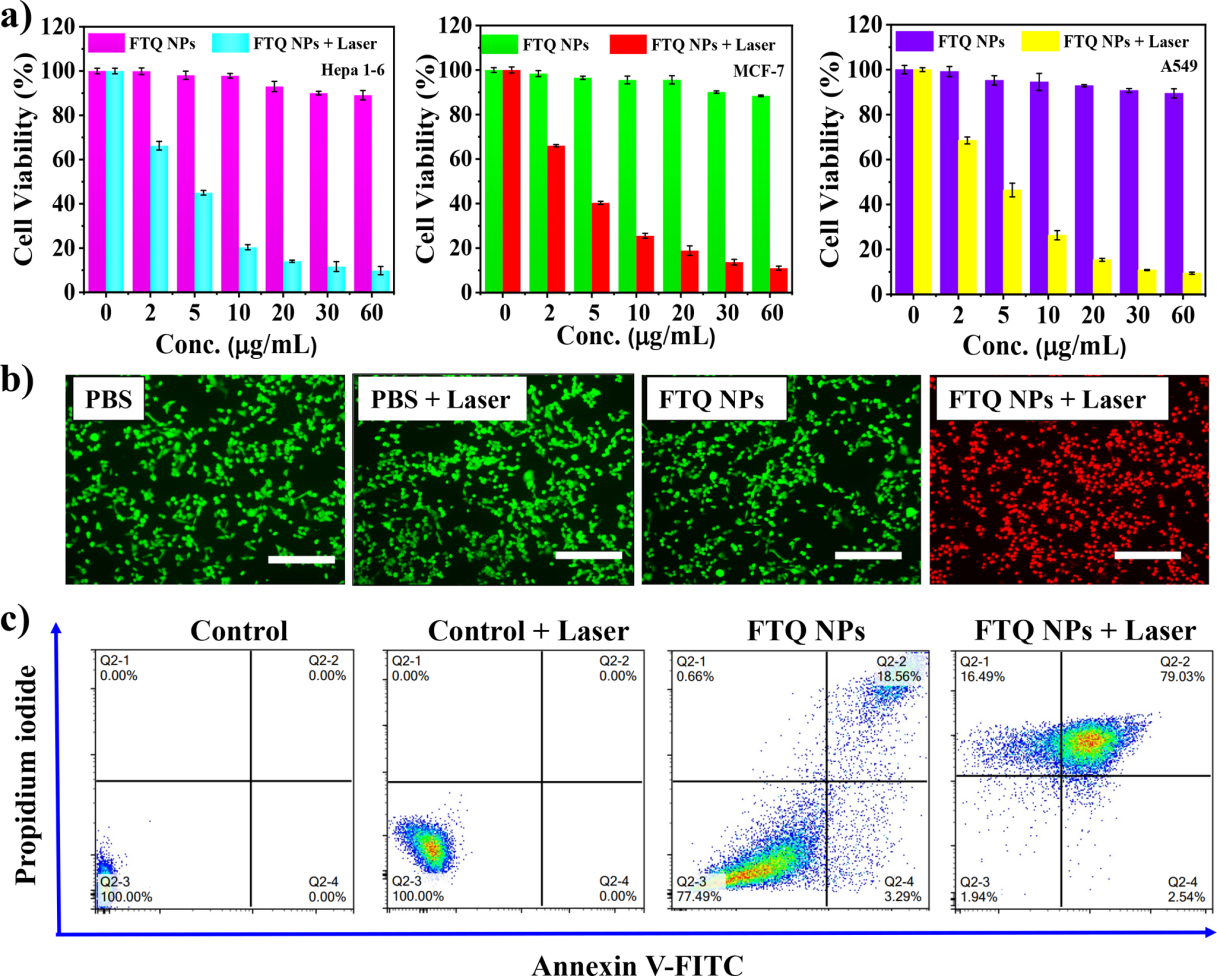
(a) Cell viabilities of Hepa 1-6, MCF-7, and A549 cells incubated with **FTQ** NPs at various concentrations with/without laser irradiation (1064 nm, 1.0 Wcm^–2^, 10 min). (b) Calcein AM (green) and propidium iodide (red) costained fluorescence images of Hepa 1-6 cells after different treatments. Scale bar: 125 μm. (c) Apoptosis/necrosis analysis by flow cytometry using Annexin V-FITC and propidium iodide.

The dark cytotoxicity and *in vitro* PTT effect of **FTQ** NPs were evaluated in different cancer cells, including Hepa 1-6, MCF-7, and A549 cell lines (Figure 4a). The cell viability was examined by using a Cell Counting Kit-8 (CCK-8) assay after incubation with **FTQ** NPs at different concentrations for 24 h. All three cell lines exhibited cell viabilities higher than 85% at NP concentrations ranging from 0 to 60 μg/mL which implied the negligible dark cytotoxicity of **FTQ** NPs. **FTQ** NPs exhibited a good *in vitro* PTT effect in the NIR-II region. When the cells were irradiated with laser light (1064 nm, 1.0 W cm^–2^) for 10 min, the cell viabilities dropped distinctly and proportionally to the NP concentrations. As shown in Table S3, the half-maximal inhibitory concentration (IC_50_) of **FTQ** NPs under irradiation in Hepa 1-6 cells is determined to be 2.21 μg/mL. Further, the cancer cell ablation ability was visualized by staining Hepa 1-6 cells with calcein acetoxymethyl (green fluorescence; living cells) and propidium iodide (red fluorescence; dead cells) as presented in Figure 4b, which confirmed the good ablation ability of **FTQ** NPs. An apoptosis/necrosis assay using Annexin V-FITC and propidium iodide was carried out to analyze the cell death by flow cytometry (Figure 4c). After NIR-II laser irradiation, **FTQ** NPs induced 2.5% of the early apoptotic cells, 16.5% of the necrotic cells, and 79.0% of the late apoptotic/necrotic cells. In contrast, neither **FTQ** NPs nor NIR-II laser irradiation alone inhibited cell growth. These results confirmed the efficient *in vitro* PTT effect of **FTQ** NPs. However, the absence of targeting functional groups in **FTQ** NPs has to be noted. The following *in vivo* PTT was performed on liver cancer, as the liver is the preferred localization for nanoparticle accumulation. Moreover, the EPR effect can additionally promote accumulation of **FTQ** NPs within the vascularized regions of tumors.

*In vivo* biotoxicity was evaluated by monitoring the long-term toxicity in healthy mice after the intravenous injection of **FTQ** NPs. The major organs (heart, liver, spleen, lung, and kidney) of mice were excised at different times (preinjection and 7 and 14 days postinjection) and processed for pathological examination with hematoxylin and eosin (H&E) staining. As shown in Figure S11, no distinct histological damages of major organs were observed when compared with the preinjection organs. At the same time, serum biochemistry and complete blood panels were studied (Figure S12), which displayed a negligible difference between preinjection and postinjection in mice. Combining these with the test results in organs and blood, the good systemic safety of **FTQ** NPs was thus confirmed.

**Figure 5:**
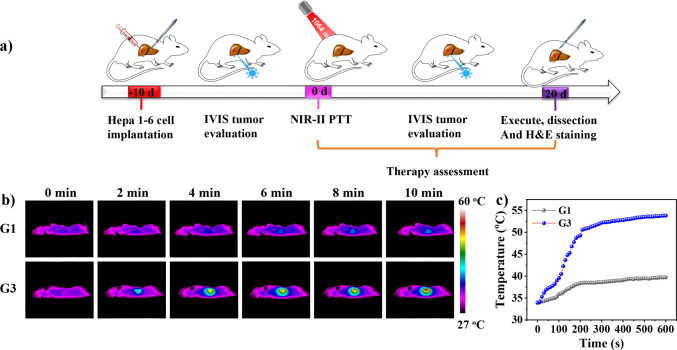
(a) Schematic illustration of the experimental procedure when applying **FTQ** NPs in PA imaging-guided PTT. IVIS: *in vivo* imaging system. (b) Thermal images of mice in G1 and G3 under 1064 nm laser irradiation (1.0 W cm^–2^) for 10 min. (c) Temperature profiles of the tumor site in G1 and G3 as a function of irradiation time.

Encouraged by the good PCE, stability, biocompatibility, and PA imaging capability, *in vivo* studies of **FTQ** NPs for PA imaging-guided PTT were conducted under 1064 nm laser irradiation. Female BALB/c mice (6 weeks old) were chosen as the Hepa 1-6 cell orthotopic liver tumor model and randomly divided into three groups with five mice in each group. The mice injected only with PBS were selected as the control group (G1) whereas the other two groups were injected with **FTQ** NPs (60 μg/mL, 100 μL) and treated without (G2)/with (G3) laser irradiation (1.0 W cm^–2^, 10 min). The experimental procedure for G3 is displayed in Figure 5a. The mice were injected with **FTQ** NPs and subjected to laser irradiation on the first day followed by a 20-day period for therapy assessment. The high-resolution PA images were obtained for tumor-bearing mice, which determined the optimum time for applying laser irradiation as 4 h postinjection (Figure 3f). The liver site temperature was recorded with an infrared thermal camera. As shown in Figure 5b, in comparison with G1, G3 displayed a distinct temperature increase to 53.5 °C (Δ*T* = 19.5 °C) thath demonstrated an efficient *in vivo* photothermal conversion of **FTQ** NPs.

**Figure 6:**
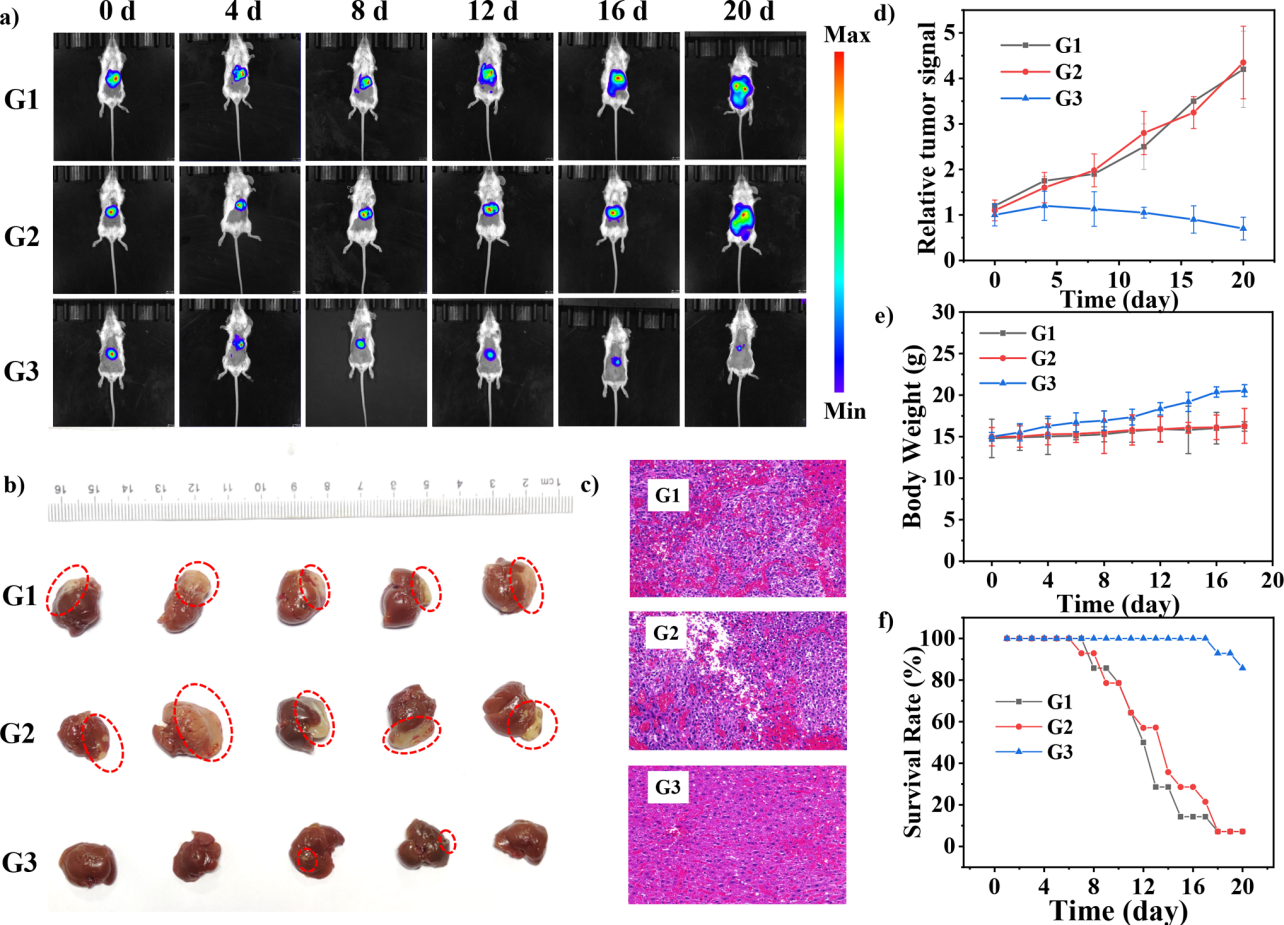
(a) Bioluminescence images of the mice in different treatment groups on days 0, 4, 8, 12, 16, and 20, respectively. (b) Excised orthotopic tumor-bearing livers and (c) representative H&E stained images of the livers after treatment for 20 days. (d) Liver tumor growth curves, (e) body weight changes, and (f) survival rates of mice in each treatment group.

To facilitate the noninvasive visualization of tumor size, the liver tumor cells were pretransfected with luciferase. During the 20 day period, bioluminescence signals from luciferase-labeled tumor sites (Figure 6a) were monitored. The tumor volumes were evaluated by measuring the luminescence radiance with an *in vivo* imaging system. As shown in Figure 6d, the tumors in G1 and G2 revealed high growth rates. On the contrary, in G3, no tumor outburst was observed after initial elimination, and the survival rates (Figure 6f) remained stable for 20-days, suggesting the inhibitory effect of **FTQ** NPs on liver tumor proliferation. Following different treatments, the liver organs of mice in G1–G3 were excised. As displayed in Figure 6b, the livers in G3 exhibited a healthy red color with minimum tumor areas among all three groups, consistent with *in vivo* bioluminescence imaging (Figure 6a). The tumor apoptosis was further evaluated via H&E staining (Figure 6c). The tumor in G3 showed a distinct necrotic region after treatment, whereas other groups exhibited a negligible change. These results demonstrated the excellent tumor ablation performance of **FTQ** NPs in PA imaging-guided PTT in the NIR-II window.

## Conclusions

In conclusion, we have developed a novel NIR-II PTT agent, **FTQ**, which exhibits NIR-II absorption with λ_max_ at 1140 nm with a tail reaching 1500 nm. Moreover, **FTQ** with ICT gives rise to a high dipole moment of 14.4 ± 0.4 Debye. Owing to the dominant nonradiative vibrational relaxation and good photostability, **FTQ** NPs deliver a high PCE of 49% under 1064 nm laser irradiation. *In vitro* and *in vivo* studies validate the promising biocompatibility, photothermal therapeutic efficacy, and PA imaging capability of **FTQ** NPs. Consequently, PTT of orthotopic liver cancer based on **FTQ** NPs can be successfully implemented in the NIR-II region guided by PA imaging. We expect that **FTQ** can be applied in multimodality synergistic theranostics in the future. At the same time, the synthetic approach will be applied to the development of new NIR-II absorbers with even larger bathochromic shifts of absorption.

## Supporting Information Available

Detailed experiment procedures and supporting figures (PDF)
