# Supplementary material for: A Terrylene–Anthraquinone Dyad as a Chromophore for Photothermal Therapy in the NIR-II Window
Source: J Am Chem Soc. 2023 Nov 27;145(48):26487–93. doi: 10.1021/jacs.3c11314 (PMC10704552; doi:10.1021/jacs.3c11314)
Supplement: Supplementary file 1 — ja3c11314_si_001.pdf [file ja3c11314_si_001.pdf]

Supporting Information

A Terrylene-Anthraquinone Dyad as Chromophore for Photothermal Therapy in the NIR-II Window

Ze-Hua Wu[†,§], Min Peng[‡], Chendong Ji[‡], Panagiotis Kardasis[#], Ioannis Tzourtzouklis[#], Martin Baumgarten[†], Hao Wu[†], Thomas Basché[§], George Floudas[#,∇,†]*, Meizhen Yin[‡]*, and Klaus Müllen[†,§]*

[†] Max Planck Institute for Polymer Research, Ackermannweg 10, 55128 Mainz, Germany

[‡] State Key Laboratory of Chemical Resource Engineering, Beijing Laboratory of Biomedical Materials, Beijing University of Chemical Technology, 100029 Beijing, China

[§] Department of Chemistry, Johannes Gutenberg-University, 55099 Mainz, Germany

[#] Department of Physics, University of Ioannina, 45110 Ioannina, Greece

[∇] University Research Center of Ioannina (URCI) - Institute of Materials Science and Computing, 45110 Ioannina, Greece

Table of Contents

1. General Methods S1

2. Synthesis details S1

3. Dipole moment measurement S4

4. Preparation of nanoparticles S7

5. Photothermal conversion efficiency (PCE) S8

6. Experiments on cells S8

7. Experiments on mouse model S10

8. Supplementary figures S11

9. NMR and Mass spectra S16

10. References S20

1. General Methods

Instrument: All reactions of air- or moisture- sensitive compounds were carried out under argon atmosphere using standard Schlenk line techniques. Nuclear Magnetic Resonance (NMR) spectra were recorded in deuterated solvents using Bruker AVANCE III 400, Bruker AVANCE III 500 or Bruker AVANCE III 700 MHz NMR spectrometers. The ^{1}H and ^{13}C chemical shifts (δ) were recorded in parts per million and the TMS signal was used as an internal standard. Coupling constants (J) were recorded in Hertz with multiplicities explained by the following abbreviations: s = singlet, d =doublet, t =triplet, dd =double of doublets, m =multiplet, br=broad. Melting points were determined on a Büchi hot stage apparatus. High-resolution mass spectra (HRMS) were recorded by atmospheric pressure chemical ionization (APCI) on a MicrOTOF-QII instrument and by matrix-assisted laser decomposition/ionization (MALDI) using 7,7,8,8-tetracyanoquinodimethane (TCNQ) as matrix on a Bruker Reflex II-TOF spectrometer. UV-vis-NIR absorption spectra were measured on a Perkin-Elmer Lambda 900 spectrophotometer at room temperature. Fluorescence spectra were recorded by two fluorescence spectrophotometers (Horiba Jobin Yvon FluoroMax-4 NIR and Edinburgh Instruments FLS980) at room temperature. Dielectric spectroscopy was measured using a Novocontrol Alpha frequency analyzer (with a frequency range from 10^{-2} to 10^{7} Hz). A 1064 nm laser (Stone-laser LTD, Beijing, China) was used in photothermal irradiation. The temperatures of samples were recorded by an IR-thermal camera (Ti400, Fluke, USA). Transmission electron microscopy (TEM, JEM-3010, JEOL, Japan) images of nanoparticles were obtained on air-dried carbon-coated copper grids. Dynamic light scattering (DLS) was measured with a Malvern Zetasizer Nano instrument with compatible disposable capillary cell (DTS 1070 from Malvern). EVOS™ FL Imaging System was used to acquire confocal microscopic images. *In vivo* fluorescence imaging was conducted utilizing IVIS® Spectrum *in vivo* imaging system (PerkinElmer Inc., Waltham, Massachusetts). Multispectral optoacoustic tomography (MSOT) INVISIO-256 system (iThera Medical) was used in a phantom and *in vivo* imaging.

2. Synthesis details

Materials: All chemical reagents and solvents were purchased from Aldrich, Acros, ABCR, TCI and used as received without further purification unless otherwise noted. Thin layer chromatography (TLC) was performed on silica gel-coated aluminum sheets with F254 indicator and column chromatography separation was performed with silica gel (particle size 0.063-0.200 mm). N-(2,6-Diisopropylphenyl)-1,6-bis[(4-(tert-butyl)phenyl)thio]-9-bromoperylene-3,4-dicarboximide (compound 1) and N-(2,6-Diisopropylphenyl)-1,6-bis[(4-(tert-butyl)phenyl)thio]-9-(5-nitronaphth-1-yl)perylene-3,4-dicarboximide (compound 2) were synthesized following the reported method.[1,2]

N-(2,6-Diisopropylphenyl)-1,6-bis[(4-(tert-butyl)phenyl)thio]-11-nitroterrylene-3,4-dicarboximide (compound 3)

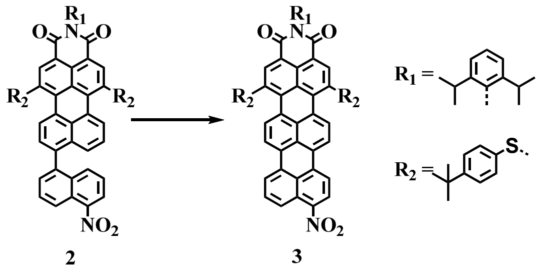

In a 25 mL Schlenk flask, compound 2 (980.0 mg, 1.0 mmol), 1,5-diazabicyclo[4.3.0]non-5-ene (1.24 g, 10.0 mmol), and sodium tert-butoxide (480.0 mg, 5.0 mmol) were dissolved in diglyme (7 mL) under N_2 atmosphere. The mixture was then heated at 70 °C for 2 hours. After cooling, the mixture was poured into 100 mL H_2O and collected through filtration. The crude product was then purified using silica column chromatography with dichloromethane/hexane (2:1) as eluent. The product was obtained as a blue solid in a yield of 70%. ^{1}H NMR (400 MHz, CD_2Cl_2, 273 K, ppm): δ 8.46 (dd, J=8.4 Hz, 2H), 8.26 (s, 1H), 8.25 (m, 2H), 8.23 (dd, J=8.4 Hz, 2H), 8.14 (d, J=8.4 Hz, 1H), 8.00 (d, J=8.4Hz, 1H), 7.92 (d, J=8.4Hz, 1H), 7.48 (t, J=8.4Hz, 1H), 7.35 (m, 8H), 7.19 (d, J=8.4Hz, 2H), 2.60 (m, J= 6.8 Hz, 2H), 1.17 (s, 18H), 0.97 (d, J=6.6Hz, 10H). ^{13}C NMR (400 MHz, CD_2Cl_2, 273 K, ppm): δ 163.34, 163.32, 152.78, 152.76, 146.04, 145.47, 136.67, 136.38, 135.63, 134.02, 133.86, 133.56, 133.44, 133.23, 133.20, 131.26, 131.13, 131.06, 130.23, 130.19, 129.89, 129.77, 129.43, 129.24, 129.11, 128.81, 128.21, 127.91, 126.95, 126.94, 126.29, 126.19, 124.89, 124.71, 123.90, 123.57, 122.72, 121.98, 121.04, 120.32, 120.08, 119.88, 34.65, 30.83, 29.01, 23.62, 23.60. HRMS (APPI+) *m/z*: calcd for $C_{64}H_{54}N_2O_4S_2$ $[M]^+$: 978.353; Found: 978.332.

N-(2,6-Diisopropylphenyl)-1,6-bis[(4-(tert-butyl)phenyl)thio]-11-aminoterrylene-3,4-dicarboximide (NH_2-TMI)

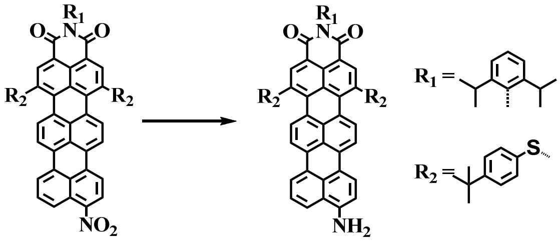

NH2-TMI

In a 50 mL Schlenk flask, compound 3 (400.0 mg, 0.41 mmol) and Pd/C (40 mg, 10 wt %) were added in ethanol (6 mL) and chloroform (3 mL). The mixture was stirred at room temperature under hydrogen pressure (balloon) for 24 hours after three vacuum/H_2 cycles to replace air with hydrogen. Then the mixture was filtered through a Büchner funnel. The product was a green solid in a yield of 78% which was used in the next step without further purification. HRMS (APPI+) *m/z*: calcd for $C_{64}H_{56}N_2O_2S_2$ $[M]^+$: 948.378; Found: 948.350.

N,N'-Bis[11-(N-(2,6-diisopropylphenyl)-1,6-bis(4-(tert-butyl)phenyl)thio-terrylene-3,4-dicarboximide)yl]-1,4-diaminoanthraquinone (compound TQ):

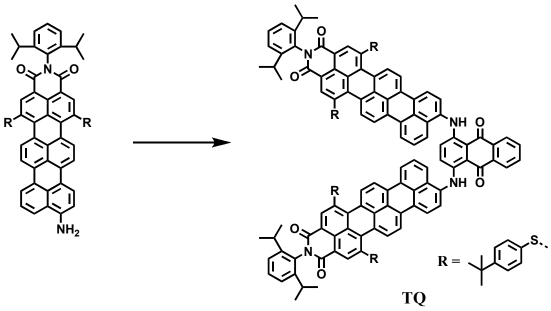

TQ

In a 100 mL Schlenk flask, NH_2-TMI (300.0 mg, 0.31 mmol), 1,4-dichloro-9,10-anthracenedione (28.0 mg, 0.1 mmol), caesium carbonate (97.5 mg, 0.35 mmol), Brettphos (0.005 mmol) and BrettPhos Pd G1 methyl t-butyl ether adduct (0.005 mmol, 0.5 mol % Pd) were added in toluene (30 mL) under N_2 atmosphere. The mixture was heated to 110 °C with stirring for 12 h. After cooling to room temperature, water (100 mL) was added. The aqueous layer was extracted with dichloromethane (100 mL) three times. The organic phases were combined, washed with brine, dried over $MgSO_4$ and evaporated. The crude product was then purified by a preparative GPC column with tetrahydrofuran as eluent. The final product was obtained as a green solid (200 mg, 70%). ^{1}H NMR (500 MHz, $C_2D_2Cl_4$, 403 K, ppm): δ 11.89 (s, 2H), 8.87 (d, J=8.0 Hz, 2H), 8.63 (s, 2H), 8.55 (d, J=8.0 Hz, 2H), 8.43 (s, 2H), 8.40 (d, J=8.0 Hz, 4H), 8.34 (d, J=8.0 Hz, 2H), 8.28 (d, J=8.0 Hz, 2H), 8.18 (d, J=8.0 Hz, 2H), 7.80 (br, 4H), 7.70 (t, J=8.0 Hz, 2H), 7.60 (m, 8H), 7.42 (m, 6H), 7.30 (m, 12H), 2.77 (m, J=6.5 Hz, 4H), 1.31 (ss, 36H), 1.15 (d, J=6.5 Hz, 24H). ^{13}C NMR (500 MHz, $C_2D_2Cl_4$, 373 K, ppm): δ 184.38, 163.33, 152.35, 145.86, 137.50, 134.72, 134.46, 133.15, 132.81, 132.65, 132.61, 131.98, 131.42, 130.75, 129.84, 129.07, 127.67, 127.47, 127.12, 126.72, 126.60, 126.55, 125.57, 123.80, 123.24, 120.10, 119.67, 113.57, 31.11, 31.08, 29.13, 23.77, 23.75. HRMS (MALDI+) *m/z*: calcd for $C_{142}H_{116}N_4O_6S_4$ $[M]^+$: 2100.7778; Found: 2100.7791.

N,N'-Bis(11-N-(2,6-diisopropylphenyl)-1,6-bis(4-(tert-butyl)phenyl)thio-terrylene-3,4-dicarboximide)-a,b-1,4-diaminoanthraquinone (FTQ):

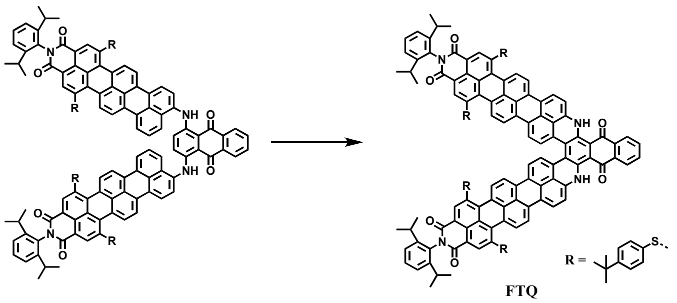

FTQ

Under N_2 atmosphere, a 25 mL Schlenk tube was charged with sodium tert-butoxide (144.0 mg, 1.5 mmol), 1,5-diazabicyclo[4.3.0]non-5-ene (248.0 mg, 2.0 mmol), and TQ (50.0 mg, 0.25 mmol) in anhydrous diglyme (5 mL). The mixture was then heated at 130 °C for 16 h. After cooling to room temperature, the crude product was

precipitated with 50 mL water and collected through filtration. The crude product was purified by a preparative GPC column with tetrahydrofuran as eluent. The final product was obtained as a grey solid in a yield of 60 % (30 mg). ^{1}H NMR (500 MHz, $C_2D_2Cl_4$, 403 K, ppm): δ 12.32 (br, 2H), 8.49-7.23 (m, 46H), 2.70 (br, 4H), 1.32-1.05 (m, 60H). HRMS (MALDI+) *m/z*: calcd for $C_{142}H_{112}N_4O_6S_4$ $[M]^+$: 2096.7465; Found: 2096.7519. Elem. Anal. Calcd for $C_{142}H_{112}N_4O_6S_4$: C, 81.27; H, 5.38; N, 2.67, Found: C, 80.30; H, 5.72; N, 2.49. IR (KBr, cm^{-1}): 3429 w, 2963 m, 1702 s, 1662 s, 1549 m, 1491 m, 1241 m, 1061 m.

3. Dipole moment measurement

Dielectric Spectroscopy (DS). The electric dipole moments were experimentally measured using dielectric spectroscopy. A Novocontrol Alpha frequency analyzer (with a frequency range from 10^{-2} to 10^7 Hz) at 20ºC was employed. DS measurements were carried out in the usual parallel plate geometry of electrodes of 20 mm in diameter and a sample thickness of 100 μm (Teflon spacers). The complex dielectric permittivity $\varepsilon^* = \varepsilon' - i\varepsilon''$ (where ε' is the real and ε'' is the imaginary part) was measured as a function of the solute concentration in chloroform. Employing the modified Onsager equation according to Böttcher and assuming ideal solutions of the two components, the dipole moment of the solute could be obtained from the derivative of the real part of the measured dielectric permittivity, ε_{12}, with respect to the concentration at the limit of infinite dilution.[3-7]

In the case of both polar solute and solvent, according to the Böttcher equation, the dielectric permittivity is given as:

$$\varepsilon_{12} = 1 + A_1\mu_1^2 + A_2\mu_2^2 + B_1R_1 + B_2R_2 \qquad (1)$$

$$A_i \equiv \frac{4\pi}{9}\frac{\varepsilon_{12}\,(2\varepsilon_{12}+1)\,(n_i^2+2)^2}{(2\varepsilon_{12}+n_i^2)^2}\frac{N_i}{k_BT}, \quad i = 1,2$$

$$B_i \equiv 3\frac{\varepsilon_{12}(n_i^2+2)}{2\varepsilon_{12}+n_i^2}\frac{N_i}{N_A}, \quad i = 1,2$$

where ε_{12} refers to the dielectric permittivity of the solution, N_i is the number density of dipoles ($N_i = \left(\frac{\rho_i}{M_i}\right)N_A$; ρ is the mass density and M is the molar mass), μ_i is the dipole moment, n_i is the refractive index and R_i is the molecular refraction in the limit of infinite wavelength ($R_i = (M_i/\rho_i) \times (n_i^2 - 1)/(n_i^2 + 2)$). Indexes 1 and 2 stand for the solute and the solvent, respectively.

The measured dielectric permittivity as a function of concentration is shown in Figure 1. Employing the slope at infinity solution, the derivative of ε_{12} (**eq. 1**), the dipole moments were calculated as $\mu_{TQ} = (14.0 \pm 0.6)$ Debye and $\mu_{FTQ} = (14.4 \pm 0.4)$ Debye. For the calculations, the refractive indexes were evaluated by measuring the dielectric permittivity of the samples in the bulk at very low temperatures (below 173 K). The corresponding values of the refractive indexes are $n_{TQ} = 3.10 \pm 0.08$ and $n_{FTQ} = 2.88 \pm 0.07$, respectively. The density was evaluated from XRD data as explained in detail below. The uncertainty in dipole moments is strongly dependent on the

uncertainty in density.

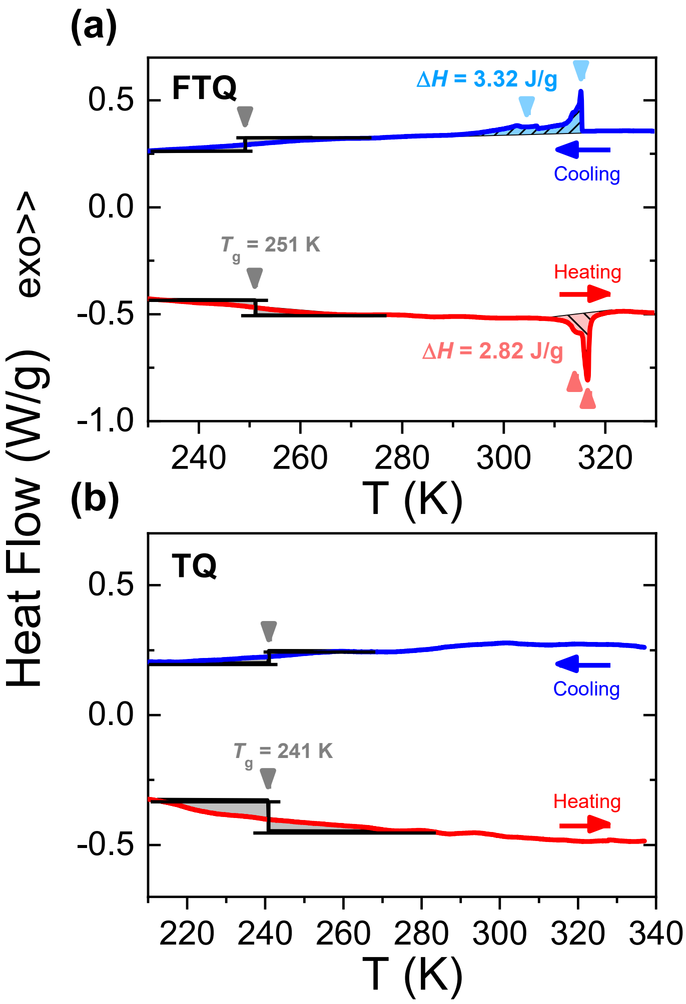

Figure S1. DSC traces of (a) **FTQ** and (b) **TQ** obtained during cooling (blue) and subsequent heating (red) at a rate of 10 K·min^{-1}. A weak crystal-melt transition is evident in **TQ**. The vertical blue and red arrows indicate the crystallization and melting points, respectively. The gray arrows indicate the liquid-to-glass transition.

Differential Scanning Calorimetry (DSC). The thermal properties of the samples were examined using differential scanning calorimetry (DSC) with a Q2000 (TA Instruments) equipped with a liquid nitrogen cooling system (LNCS) at a temperature range of 200 to 350 K. The temperature protocols involved a cooling scan and a subsequent heating scan at a rate of 10 K·min^{-1}. Samples were sealed in a Tzero aluminum low-mass pan and an empty pan was used as the reference. The instrument was calibrated in the specific temperature range for the baseline using a sapphire standard, and for the enthalpy and transition temperature employing an indium standard (ΔH = 28.71 J/g, T_m = 428.8 K, at a heating rate of 10 K·min^{-1}).
The heat flow traces of the two samples are presented in **Figure S1**. The **TQ** (precursor) is amorphous with a liquid-to-glass temperature at $T_g^{TQ} = (241 \pm 5)\,\mathrm{K}$. **FTQ** has a T_g about 10 K higher than its precursor at $T_g^{FTQ} = (251 \pm 5)\,\mathrm{K}$ associated with the more

rigid structure and the suppressed rotational freedom. In addition, a weak crystal-to-melt transition, with an enthalpy of $\Delta H = 2.82$ J/g, resembling liquid crystal transitions, is evident at a melting point $T_m^{TQ} = (317 \pm 3)$ K.

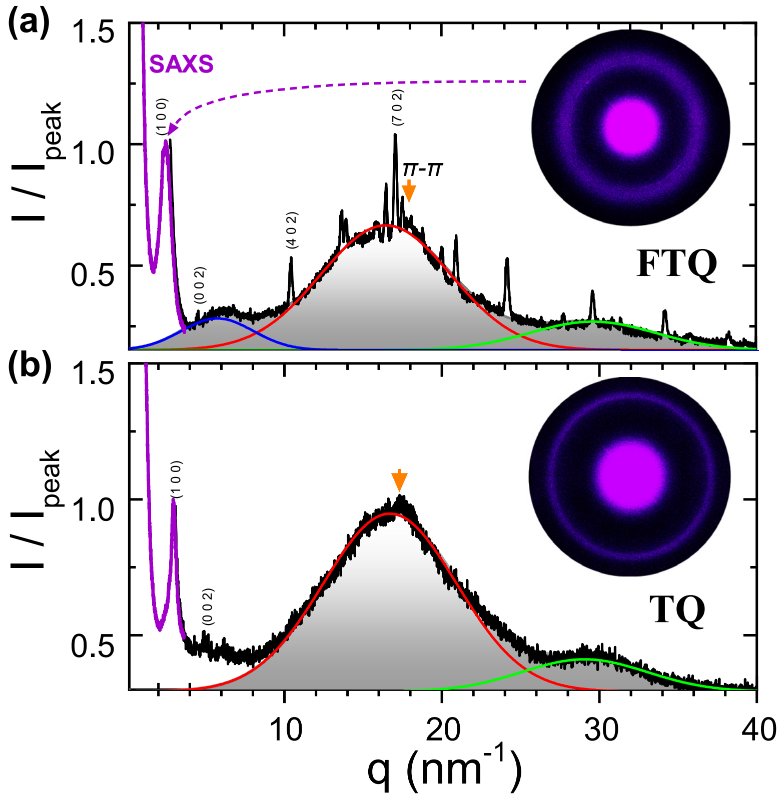

Figure S2. SAXS (purple) and WAXS (black) patterns of (a) **FTQ** and (b) **TQ** at 293.15 K. The intensities were normalized to the maximum intensity of the most pronounced peak. In the insets are presented the 2-D intensity distribution in the detector from SAXS. The blue, red and green lines indicate the deconvolution of the amorphous regions (WAXS). (a) The sample presents a low degree of crystallinity with the first Bragg reflection in the low-q (SAXS) region. (b) The sample is largely amorphous. The peak in the low-q region associates with the average packing distance.

Small-Angle X-ray Scattering (SAXS). SAXS measurements were made with the N8 Horizon vertical setup (Bruker), using a 50W CuKα radiation (IμS micro-focus source with integrated MONTEL optics). The diffraction patterns were recorded on the VÅNTEC-500 2D detector (Bruker) at a sample-detector distance of 660 mm. The samples were placed in the form of powder within borosilicate glass capillaries with a diameter of 1 mm. Intensity distributions as a function of the modulus of the total scattering vector, $q = (4\pi/\lambda)\sin(2\theta/2)$, where 2θ is the scattering angle and $\lambda = 0.154$ nm is the wavelength, were obtained by radial averaging of the 2D datasets.

Wide-Angle X-ray Scattering (WAXS). WAXS measurements were performed with a D8 Advance Bruker diffractometer, CuKα (40 kV, 40 mA) radiation, equipped with a secondary beam graphite monochromator. The system employed a Bragg-Brentano geometry in a θ–θ configuration. Patterns were obtained over the range of 2θ from 2

deg to 40 deg in steps of 0.01 deg, and the rate was 32 s per step for all samples. The recorded intensity distributions are presented as a function of the modulus of the scattering vector (λ = 1.54184 nm). Scattering curves were taken at a temperature of 293 K.

The WAXS patterns together with the SAXS patterns of the two samples are illustrated in **Figure S2**. In the case of **FTQ**, three amorphous broad regions were evident at $d = (2\pi/q) = 0.22$ nm, $d = 0.38$ nm and $d = 1.09$ nm. The first two regions were also visible in the WAXS pattern of the precursor. The peak at d = 0.22 nm, corresponds to intramolecular (bond) distances whereas the peak at d = 0.38 nm corresponds to intermolecular (van der Waals) distances. Due to the large size of **FTQ**, the first Bragg reflection was detected within the low-q (SAXS) region (q ~ 2.4 nm^{-1}). The pattern of the sharper peaks conformed to a simple orthorhombic unit cell with lattice parameters a = 2.68 nm, b = 0.35 nm and c = 2.72 nm. A few Miller indexes of characteristic peaks are shown in **Figure S2a**. The density was evaluated from the aforementioned lattice by assuming 100% crystalline material, as $\rho_{FTQ} = (1.37 \pm 0.05)\ \mathrm{g/cm^3}$. Because of the more flexible structure of **TQ**, the first peak was found at higher q (shorter distances, d) in comparison to **FTQ**. Considering that the two molecules have equivalent molecular mass, the difference suggests an increase in density. To obtain a broad estimate, we have further assumed the same orthorhombic lattice (with parameters a = 2.14 nm, b = 0.37 nm and c = 2.55 nm) and calculated the density, as $\rho_{TQ} = (1.7 \pm 0.1)\ \mathrm{g/cm^3}$. It has to be noted that there is a large uncertainty in the density estimation, especially for the precursor, **TQ**.

4. Preparation of nanoparticles

The nanoparticles of **FTQ** were prepared via a matrix-encapsulation method. In brief, a mixture of **FTQ** (1 mg) and DSPE-PEG2000 (5 mg) in DMF (2 mL) was dropped slowly into 2 mL water using a micro syringe pump. After stirring for 12 h, the micelle solution was dialyzed against deionized water via a cellulose membrane (cutoff MW: 3.5 KDa) for 48 h. The product (**FTQ** NPs) was then collected through freeze drying at 0°C. The drug loading ratio (DLR) of **FTQ** NPs was calculated to be 12.9 % according to the equation (2) as shown below:

$$\mathrm{DLR} = \frac{Weight \text{ of FTQ}}{Weight \text{ of FTQ loaded micelles}} \times 100\% \qquad (2)$$

5. Photothermal conversion efficiency (PCE)

The photothermal conversion capability of **FTQ** NPs was evaluated in water under irradiation with a 1064 nm laser (1.0 W cm^{-2}) for 10 min. The temperature changes during the experiment were monitored every 10 s by an IR thermal camera. The photothermal conversion efficiency (η) was calculated according to the following equation (3):

$$\eta = \frac{hA\Delta T_{max} - Qs}{I(1 - 10^{-A1064})} \quad (3)$$

where **h** represents the heat transfer coefficient, **A** is the surface area of the container, ΔT_{Max} represents the difference between the maximum steady-state temperature with the ambient temperature, **Qs** is the heat dissipation of solvent (water) which has been measured by a power meter (407A, Spectra-Physics), I represents the incident laser power (1.0 W cm^{-2}), and **A1064** represents the absorbance of **FTQ** NPs at 1064 nm. Herein, **hA** was calculated by the following equation (4):

$$hA = \frac{\sum m_i C_i}{\tau_s} \quad (4)$$

where m_i and C_i are the mass (1.0 g) and heat capacity (4.2 J/g) of solvent (pure water), respectively. τ_s represents the sample system time constant which was calculated by the following equation (5):

$$\tau_s = -\frac{T}{\ln\theta} \quad (5)$$

where **T** represents time. **θ** is the dimensionless driving force defined as $(T-T_{sur})/(T_{max}-T_{sur})$.

6. Experiments on cells

6.1 Cell incubation

The Hepa 1-6, MCF-7, and A549 cell lines were bought from the American Type Culture Collection (ATCC). The cells were incubated in Dulbecco's modified Eagle's medium (DMEM) added with 10% fetal bovine serum (FBS), 1% penicillin (100 unit/mL) and streptomycin (100 μg/mL) in 5% CO_2 atmosphere at 37°C.

6.2 *In vitro* cytotoxicity assay

The cytotoxicity of **FTQ** NPs was tested by the CCK-8 assay. Hepa 1-6, MCF-7, and A549 cells were cultured in 96-well plates with 6×10^3 cells per well for 24 h. The **FTQ** NPs at different concentrations (0, 5, 10, 20, 30, 60 μg/mL) were added to the cell culture medium. After another 24 h incubation, the medium was removed and washed with PBS. Subsequently, CCK-8 (10 μL) was added into each well and the cells were incubated for 4 h. The absorbance at 450 nm was then measured using a microplate reader.

6.3 Staining of dead and living cells

The Calcein-AM and PI dyes assay kit was used to stain the Hepa 1-6 cells to verify the *in vitro* photothermal effect. Cells were evenly seeded in 96-well plates at a density of 6×10^3 cells per well and were incubated for 12 h at 37 °C in 5% CO_2 atmosphere. After different treatments (incubated with/without **FTQ** NPs and irradiated), all cells were filled up with a PBS buffer (1 mL per well) containing calcein-AM (2 μM) and PI (5 μM) for 20 min in the cell-cultured container. Then, all cells in 6-well plates were washed with PBS three times and observed by fluorescence microscopy.

6.4 Photoacoustic (PA) imaging in phantoms

The multispectral optical tomography system (MSOT in Vision 256, iThera8 Medical, Germany) was used to measure PA signal. PA imaging in phantoms was measured with agar as carrier. The samples were prepared by loading the mixture of control group (DI water) or **FTQ** NPs aqueous solution at different concentrations (2, 5, 10, 20, 30 and 60 μg/mL) with agar into the plantation. The distribution of photoacoustic signal intensity was tested at different excitation wavelengths (680, 685, 690, 695, 700, 710, 730, 750, 780, 800, 850 and 900 nm) in order to obtain the optimal excitation wavelength for photoacoustic imaging.

7. Experiments on a mouse model

7.1 Tumor-bearing mouse model

All animal studies were conducted under the guidelines set by the Ethical Committee Peking Union Medical College and performed under legal protocols. Six-week-old female BALB/c mice were purchased from the Beijing Vital River Laboratory Animal Technology Co., Ltd. The tumor-bearing mouse model was established by xenotransplantation of Hepa1-6 cells. In brief, Hepa1-6 cells (1×10^7) suspended in 50 μL of PBS were injected subcutaneously into the liver part of the mouse. After 10 days, the mice were used in the subsequent experiments.

7.2 Photoacoustic imaging *in vivo*

The tumor-bearing mice were injected with **FTQ** NPs (60 μg/mL, 100 μL) through the tail vein and placed in a dark box containing a 37 °C water tank after being anesthetized with 2% isoflurane in oxygen. The PA signals at the liver tumor site were collected using a multispectral PA tomography instrument as a function of post-injection times under the maximum excitation wavelength (680 nm, laser power: 5.02 m J). The mice injected with 100 μL 1×PBS were used as blank control.

7.3 *In vivo* photothermal therapy

Female BALB/c mice (6 weeks old) were chosen as the Hepa 1-6 cells orthotopic liver cancer model and the bioluminescence intensity is 10^8. The mice were randomly divided into three groups (n = 10 per group), named as G1-G3. The mice injected only with PBS were selected as control group (G1). The other two groups were injected with **FTQ** NPs and treated without (G2)/with (G3) 1064 nm laser irradiation (1.0 W cm^{-2}) for 10 min. For G1 and G3, after intravenous injection of pure PBS or **FTQ** NPs for 4h, the liver tumor site of each mice was continuously irradiated with 1064 nm laser (1.0 W/cm^2) for 10 min. In the meantime, the temperature changes of the tumors were recorded via a Fluke IR thermal camera. After different treatment for 20 days, the mice in G1-G3 were dissected. The liver organs were excised and paraffin-embedded, sectioned and stained by hematoxylin and eosin (H&E) for histopathological evaluation.

8. Supplementary Figures

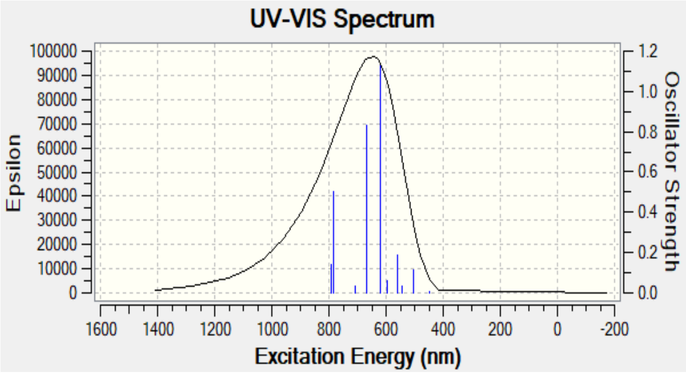

Figure S3. Simulated absorption of **TQ** from DFT calculation (B3lyp/6-31g*).

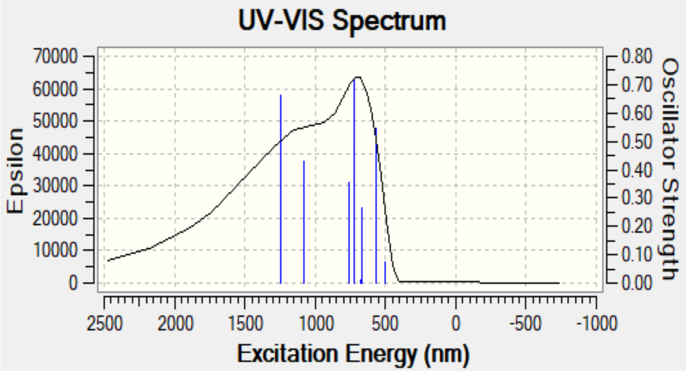

Figure S4. Simulated absorption of **FTQ** from DFT calculation (B3lyp/6-31g*).

Table S1. Calculated vertical transitions and simulated absorption parameters of **FTQ** from DFT calculation (b3lyp/6-31g*).

State	Configuration	Contribution	Excitation (eV)	Oscillator strength (f)	Wavelength (nm)[a]
**FTQ**					
S_1	H → L	100%	1.0111	0.6020	1226.25
S_2	H-1 → L	97.4%	1.1468	0.4129	1081.09
S_3	H-1 → L+1	96.4%	1.6330	0.4060	759.26
S_4	H-2 → L	14.7%	1.7232	0.6742	719.50
	H → L+1	78.1%			
S_6	H → L+2	91.2%	1.8733	0.2407	661.84
S_7	H-2 → L	48.8%	2.1681	0.4940	571.86
	H-1 → L+2	34.1%			

Table S2. Summary of electrochemical data

	HOMO[a]	LUMO[a]	E_{gap}[a]	HOMO[b]	LUMO[b]	E_{gap}[b]	E_{gap}[c]
**TQ**	-4.90	-3.12	1.78	-5.25	-3.78	1.47	1.47
**FTQ**	-4.83	-3.66	1.17	-4.56	-3.74	0.86	0.82

a) DFT calculation result (B3LYP/6-31 G*). b) Data from cyclovoltammetry using Fc/Fc+ standard (carried out in DCM containing 0.1 M n-Bu_4NPF_6 as supporting electrolyte at room temperature. A glassy carbon electrode was

used as a working electrode, a platinum wire as a counter electrode, and a silver wire as a reference electrode). c) Optical bandgap calculated from the absorption onset.

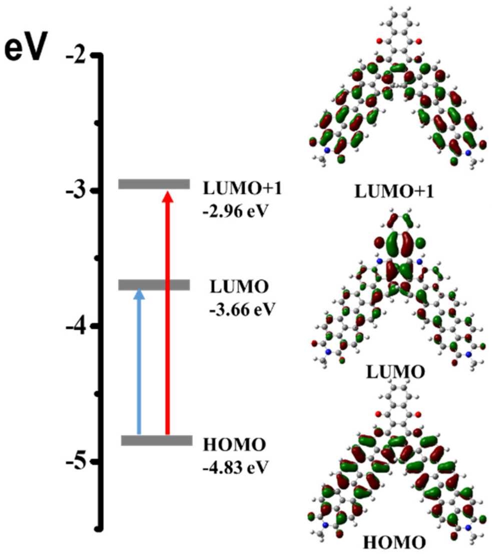

Figure S5. Molecular orbitals of **FTQ** and the energy level diagrams from DFT calculation (B3lyp/6-31g*)

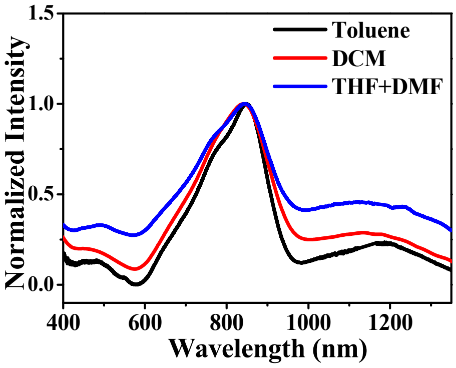

Figure S6. Absorption spectra of **FTQ** in different solvents.

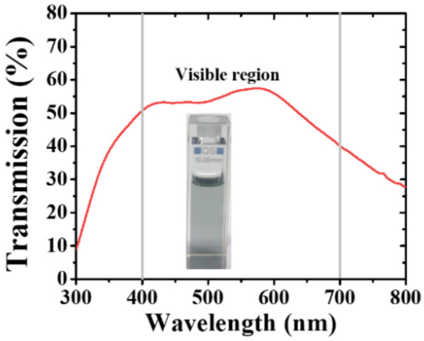

Figure S7. Transmission spectra of **FTQ** in solution (1×10^{-3} M in dichloromethane). Inset: solution of **FTQ** (1×10^{-3} M in dichloromethane).

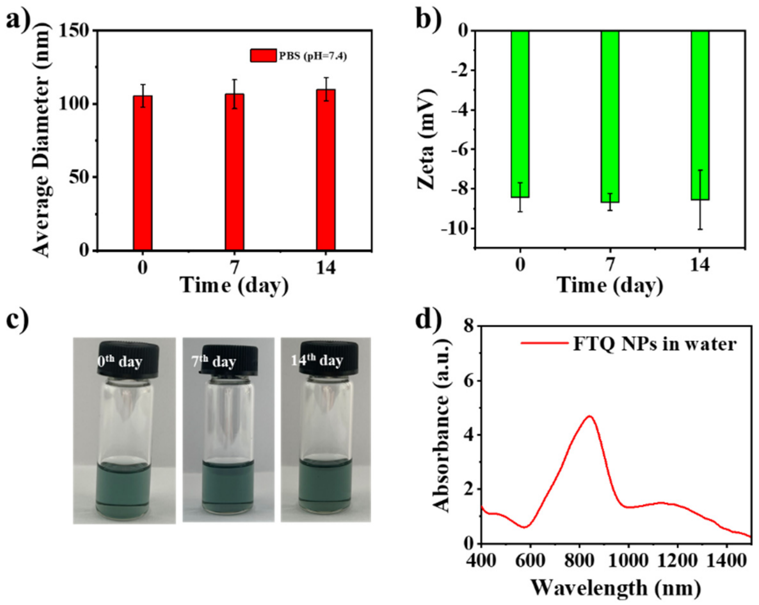

Figure S8. a) Average diameters of **FTQ** NP in PBS solutions (pH = 7.4); b) Average zeta potentials of **FTQ** NP in PBS solutions (pH = 7.4). Error bars, mean ± SD; c) Optical photographs of **FTQ** NP in PBS solutions (pH = 7.4); d) Absorption spectrum of **FTQ** NP in water.

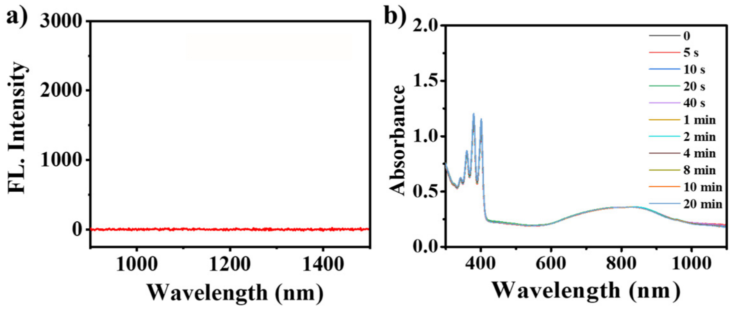

Figure S9. a) Emission spectrum of **FTQ** (5×10^{-6} M in dichloromethane); b) Absorption spectra of mixture aqueous solution of **FTQ** NPs and 9,10-anthracenediyl-bis(methylene) dimalonic acid under continuous white light irradiation (400-700 nm, 20 mW cm^{-2}).

Table S3. Photo-indexes values

λ_{max} [a] (nm)	ε [b] ($M^{-1}cm^{-1}$)	Dipole Moment (Debye)	Φ_f [c]	Particle Diameter (nm)	PCE[d]	IC_{50} [e] (μg/mL)	Laser Wavelength (nm)	Laser Intensity (Wcm^{-2})
1140	17628	14.4±0.4	-	110.8 ± 0.8	49%	2.21	1064	1.0

a) Maximum absorption peak. b) Extinction coefficient at λ_{max}. c) Fluorescence quantum yield was negligible. d) Photothermal conversion efficiency under 1064 nm laser irradiation. e) IC_{50} under irradiation was calculated using the formula: $lgIC_{50} = X_m - I[P - (3 - P_m - P_n)/4]$, where X_m is $lg(maximum\ dose)$; I is $lg(maximum\ dose/adjacent\ dose)$; P represents the sum of positive response rate; P_m represents the largest positive response rate; P_n represents the smallest positive response rate.

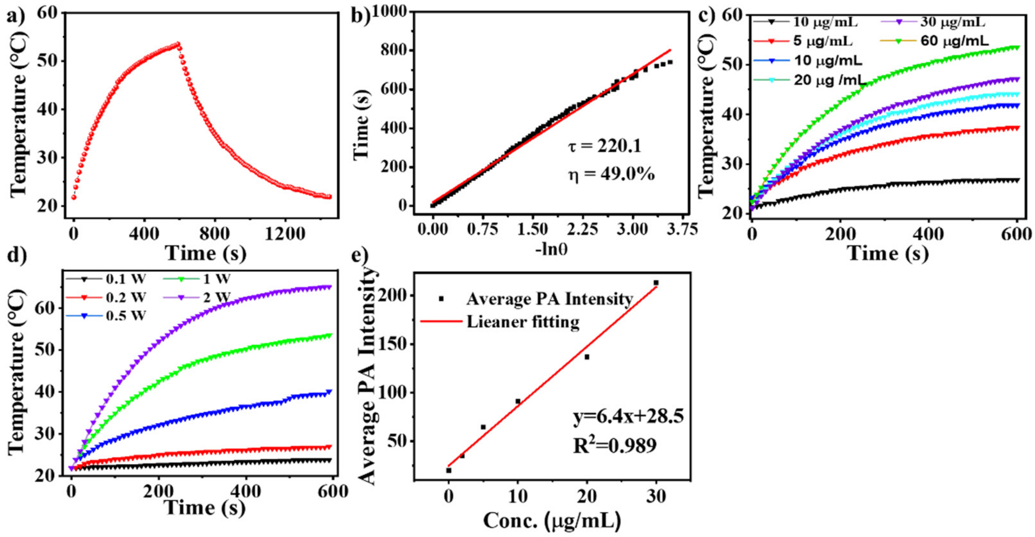

Figure S10. a) Photothermal heating curves of **FTQ** NPs (60 μg/mL) under 1064 nm irradiation (1 W cm^{-2}) for 10 min followed by cooling to room temperature. b) Linear correlation of the cooling times versus negative natural logarithm of θ. θ is the dimensionless driving force defined as ($T-T_{sur}$)/($T_{max}-T_{sur}$); c) Photothermal conversion of **FTQ** NPs under 1064 nm laser irradiation at different concentrations (0-60 μg/mL); d) Photothermal conversion of **FTQ** NPs (60 μg/mL) under 1064 nm laser irradiation with different intensity (0.1-2 W cm^{-2}); e) Average PA signal intensity as a function of nanoparticle concentration (coefficient of determination, R^2=0.99).

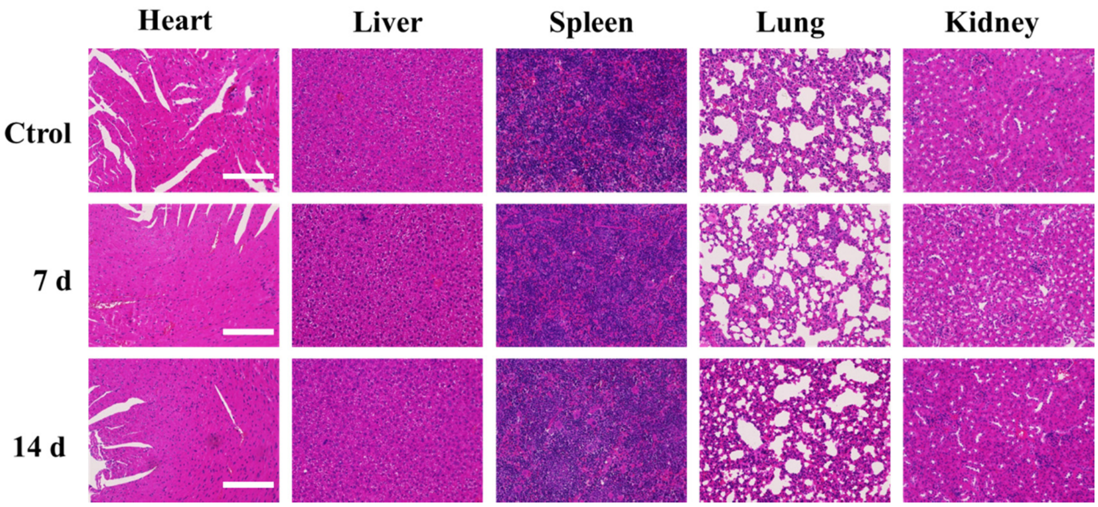

Figure S11. The pathological examination by H&E staining of major organs collected from healthy mice after injection of **FTQ** NPs via tail. Scar bar: 150 μm.

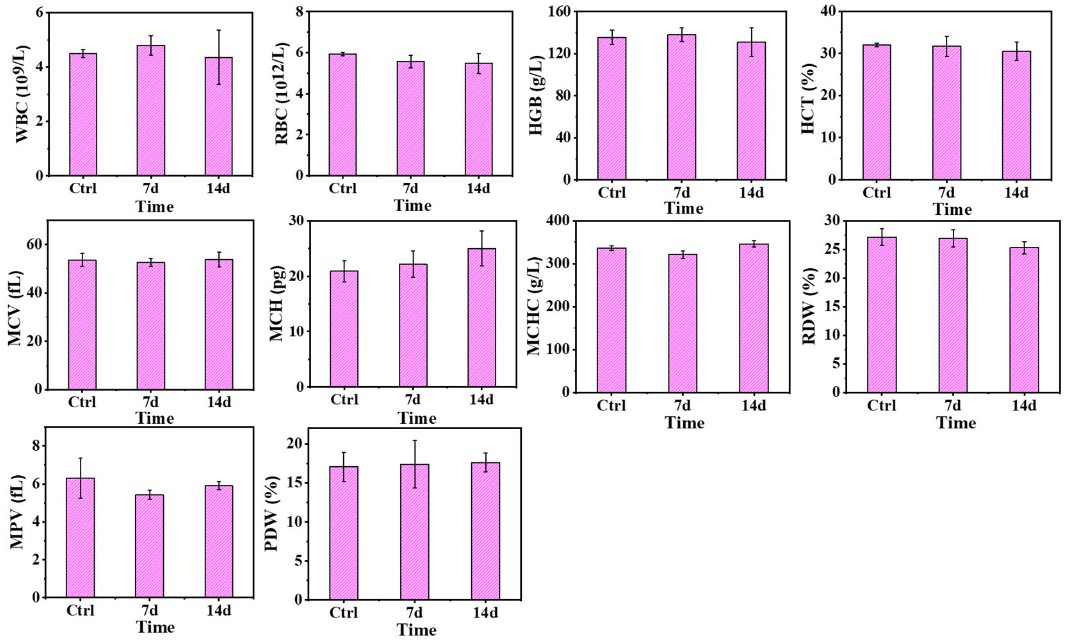

Figure S12. The serum biochemistry and complete blood panels analysis (WBC represents white blood cell, RBC represents red blood cell count, HGB represents hemoglobin test, HCT represents hematocrit test, MCV represents mean corpuscular volume, MCH represents mean corpuscular hemoglobin, MCHC represents mean corpuscular hemoglobin concentration, RDW represents red cell distribution width, MPV represents mean platelet volume, PDW represents platelet distribution width). Healthy female Balb/c mice intravenous injected with aqueous solutions of **FTQ** NPs (dose = 45 mg/kg) were sacrificed after 7 and 14 days for blood collection. Untreated healthy mice were used as control. Error bars, mean ± SD.

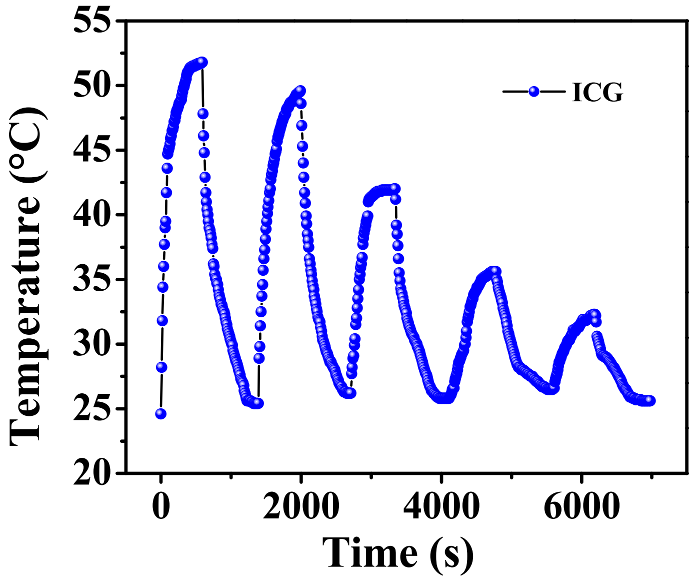

Figure S13. Photothermal stability of ICG upon 808 nm laser irradiation of 1 W cm^{-2} for five on/off cycles, serving as a comparison of **FTQ** NPs.

9. NMR and Mass Spectra

^1H NMR (400 MHz, CD_2Cl_2, 293 K) of **compound 3**

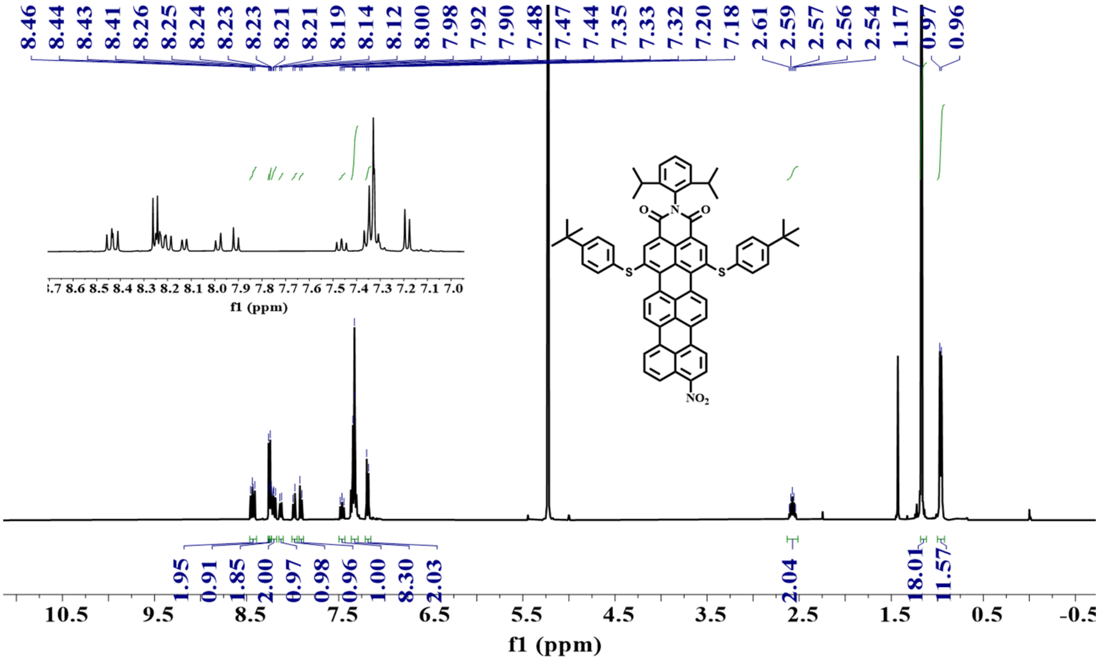

^{13}C NMR (400 MHz, CD_2Cl_2, 293 K) of **compound 3**

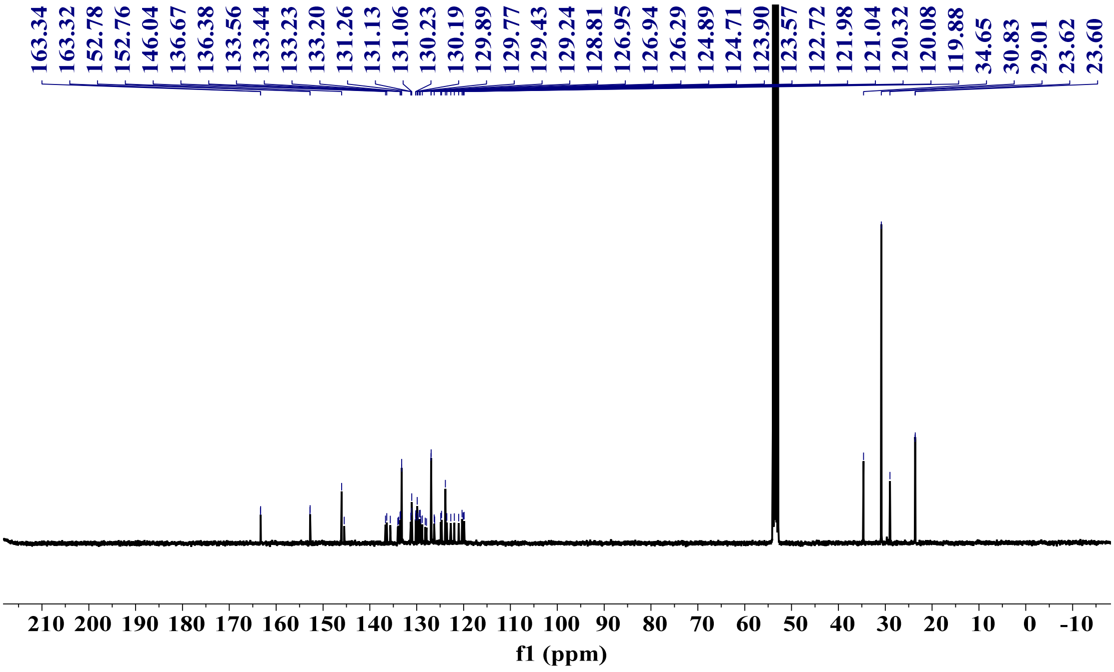

^{1}H NMR (500 MHz, CD_2Cl_4, 403 K) of **TQ**

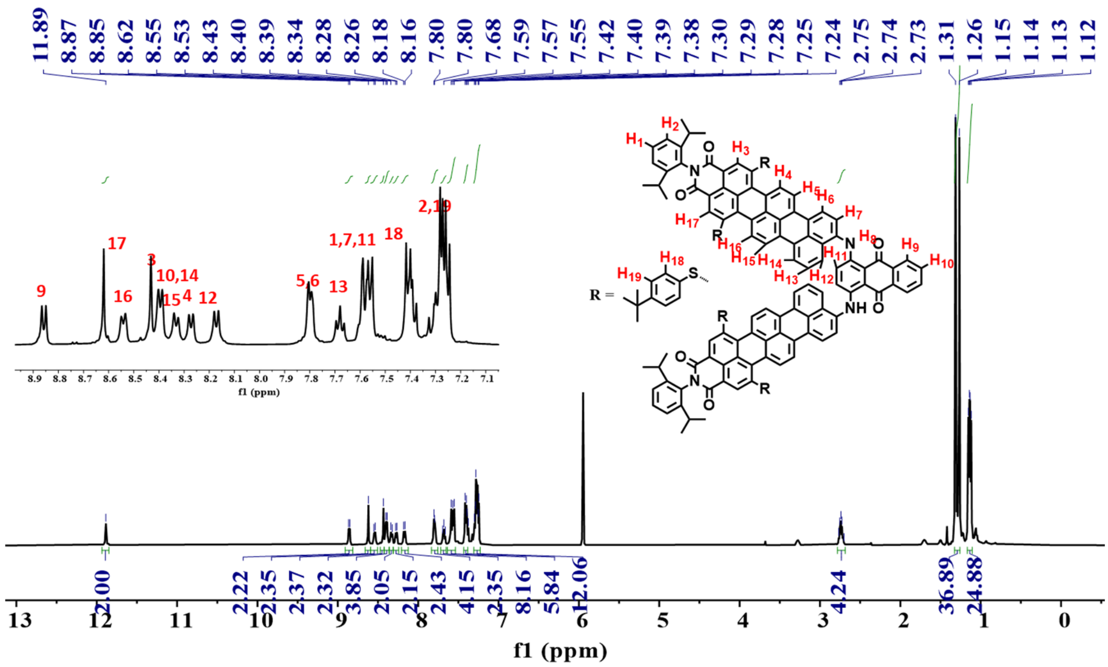

Aromatic region of the ^1H-^1H COSY spectrum of **TQ** (500 MHz, CD_2Cl_4, 403 K)

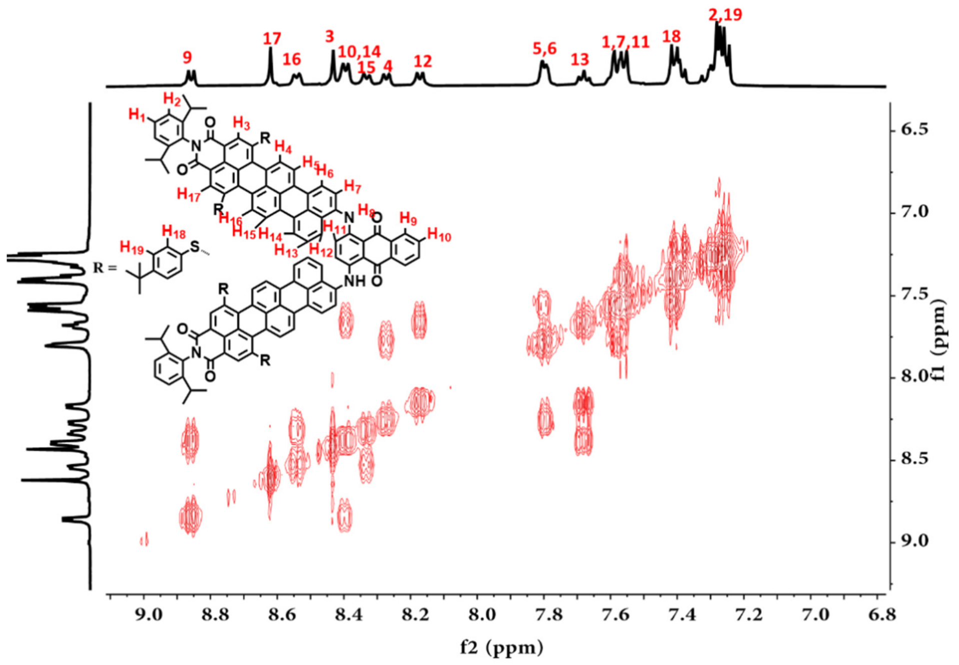

Aromatic region of the ^{1}H-^{1}H NOESY spectrum of **TQ** (500 MHz, CD_2Cl_4, 403 K)

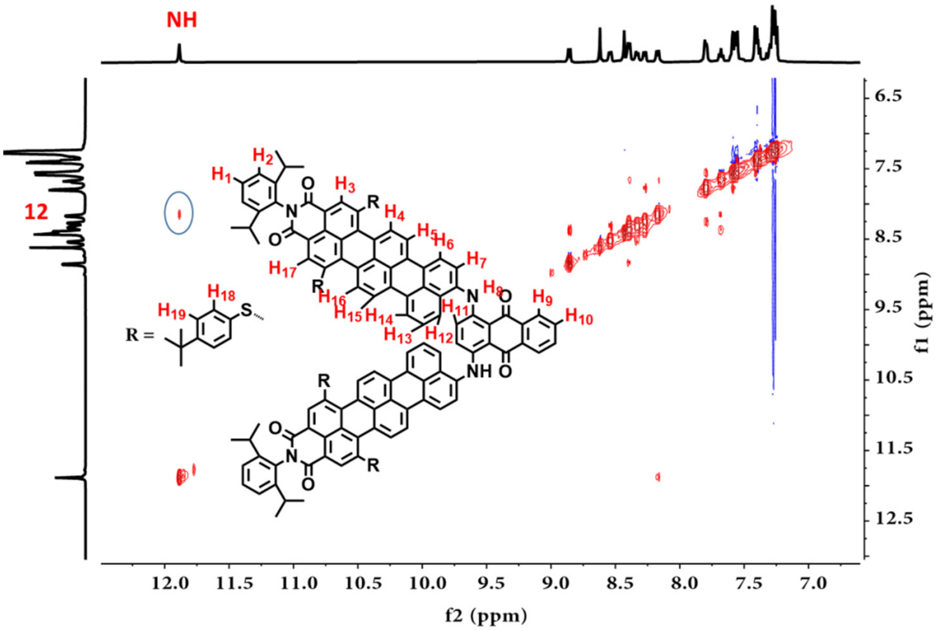

Spin-echo ^{13}C NMR (126 MHz, CD_2Cl_4, 403 K) of **TQ**

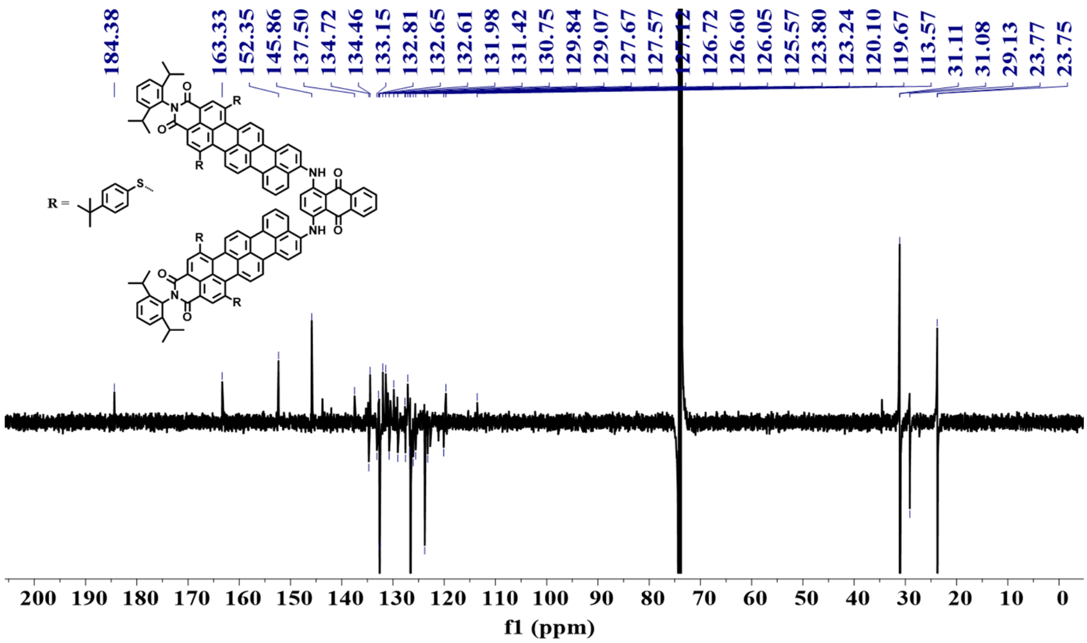

^{1}H NMR (500 MHz, CD_2Cl_4, 403K) of **FTQ**

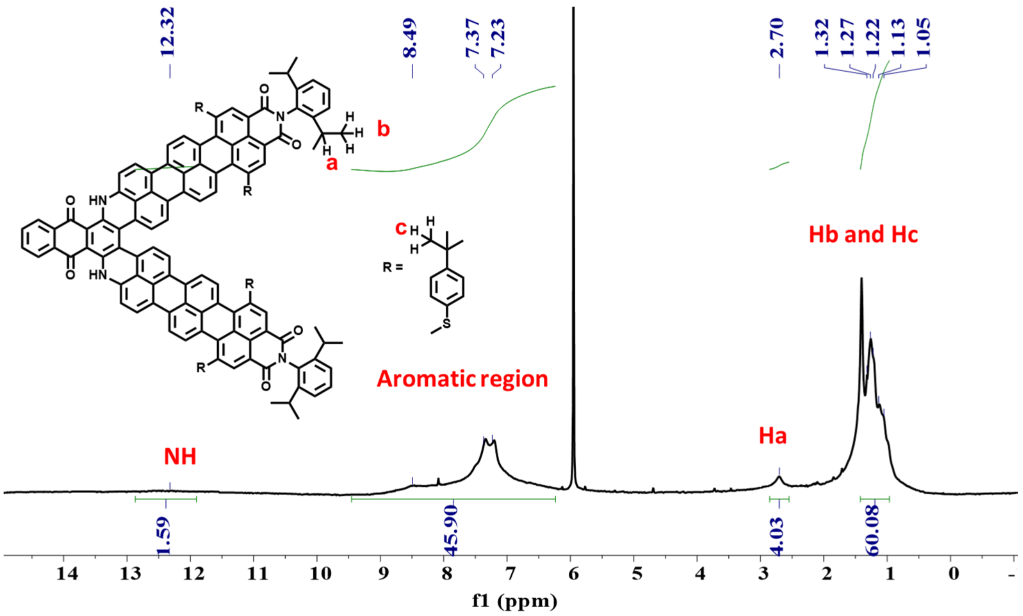

MALDI-TOF mass spectra of **TQ**

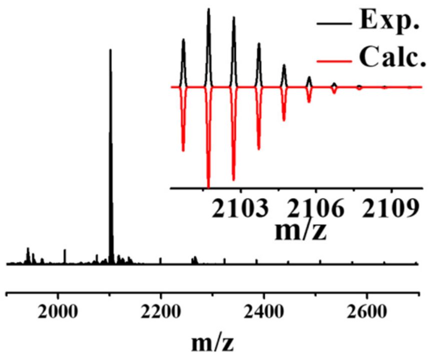

MALDI-TOF mass spectra of **FTQ**

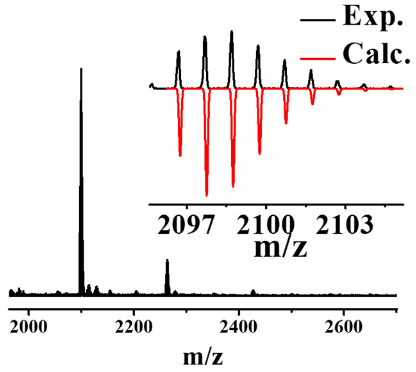

10. References

1. Sekita, M.; Jiménez, Á. J.; Marcos, M. L.; Caballero, E.; Rodríguez-Morgade, M. S.; Guldi, D. M.; Torres, T. Tuning the Electron Acceptor in Phthalocyanine-Based Electron Donor–Acceptor Conjugates. *Chem. Eur. J.* **2015**, *21*, 19028.
2. Hu, G.; Liu, R.; Alexy, E. J.; Mandal, A. K.; Bocian, D. F.; Holten, D.; Lindsey, J. S. Panchromatic chromophore-tetrapyrrole light-harvesting arrays constructed from Bodipy, perylene, terrylene, porphyrin, chlorin, and bacteriochlorin building blocks. *New J. Chem.* **2016,** *40*, 8032.
3. Böttcher, C. J. F. Eine Neue Methode zur Berechnung von Dipolmomenten. *Rec. Trav. Chim.* **1943**, *62*, 119.
4. Rizk, H.A.; Elanwar, I.M. Dipole Moments of Glycerol, Isopropyl Alcohol, and Isobutyl Alcohol. *Can. J. Chem.* **1968**, *46*, 507.
5. Wudarczyk, J.; Papamokos, G.; Margaritis, V.; Schollmeyer, D.; Hinkel, F.; Baumgarten, M.; Floudas, G.; Müllen, K. Hexasubstituted Benzenes Bearing Ultrastrong Dipole Moments. *Angew. Chem. Int. Ed.* **2016**, *55*, 3220.
6. Wudarczyk, J.; Papamokos, G.; Marszalek, T.; Nevolianis, T.; Schollmeyer, D.; Pisula, W.; Floudas, G.; Baumgarten, M.; Müllen, K. Dicyanobenzothiadiazole Derivatives Possessing Switchable Dielectric Permittivities, *ACS Appl. Mater. Interfaces,* **2017**, *9*, 20527.
